# Overcoming Perovskite Corrosion and De-Doping Through Chemical Binding of Halogen Bonds Toward Efficient and Stable Perovskite Solar Cells

**DOI:** 10.1007/s40820-022-00916-3

**Published:** 2022-08-23

**Authors:** Guanhua Ren, Wenbin Han, Qiang Zhang, Zhuowei Li, Yanyu Deng, Chunyu Liu, Wenbin Guo

**Affiliations:** State Key Laboratory of Integrated Optoelectronics, College of Electronic Science and Engineering, Jilin University, 2699 Qianjin Street, Changchun, 130012 People’s Republic of China

**Keywords:** 4-tert-butylpyridine, Corrosion, De-doping, Chemical binding, Stability

## Abstract

4-tert-butylpyridine (TBP) is an indispensable additive for the hole transport layer in highly efficient perovskite solar cells (PSCs), while it can induce corrosion decomposition of perovskites and de-doping effect of spiro-OMeTAD, which present huge challenge for the stability of PSCs. Herein, halogen bonds provided by 1,4-diiodotetrafluorobenzene (1,4-DITFB) are employed to bond with TBP, simultaneously preventing perovskite decomposition and eliminating de-doping effect of oxidized spiro-OMeTAD. Various characterizations have proved strong chemical interaction forms between 1,4-DITFB and TBP. With the incorporation of halogen bonds, perovskite film can maintain initial morphology, crystal structure, and light absorbance; meanwhile, the spiro-OMeTAD film shows a relatively stable conductivity with good charge transport property. Accordingly, the device with TBP complex exhibits significantly enhanced stability in N_2_ atmosphere or humidity environment. Furthermore, a champion power conversion efficiency of 23.03% is obtained since perovskite is no longer damaged by TBP during device preparation. This strategy overcomes the shortcomings of TBP in n-i-p PSCs community and enhances the application potential of spiro-OMeTAD in fabricating efficient and stable PSCs.

**Supplementary Information:**

The online version contains supplementary material available at 10.1007/s40820-022-00916-3.

## Highlights

- Chemical binding of 4-tert-butylpyridine (TBP) is realized through halogen bonds.
- TBP-induced perovskite decomposition and spiro-OMeTAD de-doping are suppressed.
- Modified perovskite solar cells achieve greatly improved efficiency and stability.

**Supplementary Information:**

The online version contains supplementary material available at 10.1007/s40820-022-00916-3.

## Graphical abstract

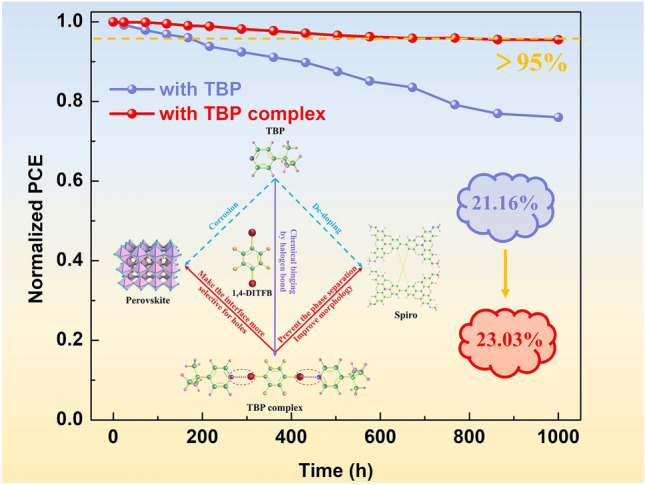

## Introduction

Organic–inorganic hybrid perovskite solar cells (PSCs) have reached a certified record power conversion efficiency (PCE) of 25.7% recently, while the large-scale commercial applications are still limited by the poor stability [1–3]. Numerous efforts have been performed to solve the stability problems caused by water, oxygen, light and heat, as well as the instability of metal oxide/perovskites interface, and several effective strategies have been provided and implemented [4–12]. Everyone knows 2,2′,7,7′-tetrakis-(N,N-di-4-methoxyphenylamino)-9,9′-spirobifluorene (Spiro-OMeTAD, abbreviated as spiro in the following) brings potential risks to stability for n-i-p PSCs [13, 14]. Additives of bis(trifluoromethane) sulfonamide lithium salt (LiTFSI) and 4-tert-butylpyridine (TBP) are needed to enable the device PCE to reach more than 25% at laboratory scale due to lower intrinsic conductivity and hole mobility of pristine spiro [15–18]. Much attention was paid to the device degradation due to the hygroscopicity and Li^+^ migration, while the effects of TBP on the stability were rarely addressed and reported at present [19–21].

Actually, TBP has been found to interact with PbI_2_, leading to corrosive decomposition of perovskite film [22, 23]. Although the utilization of TBP-LiTFSI complex with the molar ratio of 4:1 can alleviate perovskite corrosion [24], the 6:1 TBP-LiTFSI was more widely applied and the highest device performance is still based on the spiro with more TBP [17]. Moreover, TBP as a nucleophile can react with oxidized spiro (spiro^+^) to form pyridinated derivatives, which was further confirmed by Lamberti et al. in detail [25–27]. The chemical interaction between TBP and spiro^+^ induces fast de-doping and reduces the conductivity of hole transport layer (HTL). These two effects will deteriorate the efficiency and long-term stability of PSCs. However, TBP plays an essential role in suppressing photogenerated electron recombination, promoting the dissociation of LiTFSI, preventing the phase separation of HTL, making the interface more selective for holes and so on [27–29]. Though the exact role of TBP is still unclear, it is indispensable for realizing high efficiency PSCs. Introducing the barrier layer fails to completely solve the TBP-induced instability, and this strategy inevitably affects the interface hole transport.

It can be found that the TBP-induced instability arises from reactive activity of pyridine ring [22, 27]. Accordingly, we conceive to introduce an ideal chemical interaction with pyridine of TBP, thus preventing the further reaction with perovskite and spiro^+^. Halogen bond has once been established as a crucial interaction in supramolecular chemistry [30], and binding energy of halogen bond is higher than that of regular hydrogen bond [31]. Herein, the 1,4-diiodotetrafluorobenzene (1,4-DITFB) as an ideal halogen bond donor was employed to pre-interact with TBP additive to form complex, since I atoms give strong contacts and F atoms are strong electron-withdrawing groups [32], consequently preventing the corrosion of perovskite film and suppressing the de-doping of spiro^+^. We systematically characterized and analyzed the film morphology, crystallization, light absorption and photoluminescence (PL) of FA_1-x_MA_x_PbI_3_ perovskites. The electrical and chemical properties of spiro in solid and solution states have been comprehensively explored as well. Resultantly, PSCs with TBP complex achieve better performance than the devices with TBP in long term and humidity stability tests, as well as a champion PCE of 23.03%.

## Experimental Section

### Materials

The SnO_2_ colloidal dispersion in H_2_O (tin (IV) oxide, 15 wt%) was purchased from Alfa Aesar. The lead iodide (PbI_2_) (> 99.99%), FAI (≥ 99.5%), MAI (≥ 99.5%), methylammonium chloride (MACl) (> 99.5%), spiro-OMeTAD (≥ 99.8%), LiTFSI (> 99%), TBP (> 96%) and PEAI (≥ 99.5%) were purchased from Xi’an Polymer Light Technology Corp. (China). The 1,4-DITFB (97%) was obtained from Macklin. The N,N-dimethylformamide (DMF, 99.9%) and dimethyl sulfoxide (DMSO, 99%) were obtained from J&K Scientific. The isopropanol (IPA, ≥ 99.7%) was purchased from Sinopharm Chemical Reagent Co., Ltd.

### Device Fabrication

The ITO-coated glass substrates were precleaned and treated with UV-zone for 20 min. The SnO_2_ precursor was obtained by mixing the SnO_2_ colloidal dispersion and deionized water in a volume ratio of 1:4. Then, the SnO_2_ precursor was spin coated on the substrate at 3000 rpm for 30 s and then annealed at 150 °C for 20 min to obtain the SnO_2_ electron transport layer. The PbI_2_ (1.5 mmol) was dissolved in 1 mL DMF and DMSO (V/V = 9:1), and the solution was spin coated onto the cooled substrate at 2000 rpm for 30 s and then annealed at 70 °C for 1 min. Subsequently, 100 μL of organic mixture solution of (FAI:MAI:MACl = 90 mg:6.39 mg:9 mg in 1 mL IPA) was spin coated onto the PbI_2_ film at 2000 rpm for 30 s and annealed at 150 °C for 30 min to form FA_1-x_MA_x_PbI_3_ perovskite films. The PEAI solution (3 mg mL^−1^ in IPA) was dynamically spin coated on the perovskite at 4000 rpm for 30 s. 1,4-DITFB was dissolved in TBP at different mass fractions (0, 0.5, 1, 2, and 3 wt%). To obtain the spiro solution, 72.3 mg of spiro, 17.6 μL of LiTFSI solution (520 mg mL^−1^ in acetonitrile) and 28.5 μL TBP or 1,4-DITFB-added TBP were dissolved in 1 mL chlorobenzene. Next, the spiro solution was deposited at 4000 rpm for 30 s as the HTL. After that, they are placed in a drying tower for oxidation. Finally, 100-nm thick of Ag electrode was deposited in a vacuum condition (< 10^–4^ pa). The devices were completed, and the effective area is 0.044 cm^2^. In the stability tests, 80-nm thick of Au film was deposited in the same way as Ag electrode.

### Characterizations

*X-ray diffraction (XRD):* The XRD patterns of the perovskite films were measured on a Shimadzu XRD-6000 diffractometer.

*Scanning electron microscope (SEM):* The cross sectional and surface SEM images were characterized using a JEOL JSM-7500F field-emission SEM.

*Atomic force microscope (AFM):* The AFM images of the perovskite films and spiro films were obtained with an ICON-PT in tapping mode.

*Absorption spectra:* The absorption spectra of the perovskite films, spiro films and solutions were performed with UV 1700 photometer, Shimadzu.

*Photoluminescence (PL):* The PL spectra were conducted on a Shimadzu RF 5301 fluorescence spectrophotometer.

The samples used for the above measurements and shown in Figs. 2d–i and S8-S12 were prepared as the following: We prepared four perovskite films for each measurement. All perovskite films were deposited on the same SnO_2_ to maintain consistency with the complete PSCs and exclude the effect of charge transport. The “control” film represents the perovskite film without any treatment; the “with TBP” film represents that we spin-coated the spiro with TBP on the perovskite film and washed it off with CB at the end; the “with TBP complex” film represents that we spin-coated the spiro with TBP complex on the perovskite film and washed it off with CB at the end; the “CB washed” film represents the perovskite film washed with CB for comparison. The amount of CB was 50 μL and washed twice dynamically. Each sample was measured for three different time periods, the first time period is that we placed the samples in the drying tower until the end of the spiro oxidation (the perovskite films without spiro were treated in the same way), the second time period is that we stored the oxidation-completed samples in N_2_ for 3 days, and the third time period is that we stored the oxidation-completed samples in N_2_ for 7 days. The PL intensities of these samples are based on the “control” film just after oxidation as a standard “1”.

*Current–voltage (J-V):* The *J-V* curves were measured using a Keithley 2400 source meter under AM 1.5G solar illumination.

*X-ray photoelectron spectroscopy (XPS):* XPS measurements were conducted on a Thermo Scientific ESCALANTM 250Xi system.

*Fourier-transform infrared spectroscopy (FTIR):* The FTIR spectra were collected using a Nicolet iS50 FT-IR, and the mass fraction ratio of 1,4-DITFB and TBP was selected 3 wt% for the convenience of analysis.

*Electron spin resonance (ESR):* The ESR spectra were measured by an electron paramagnetic resonance spectrometer (BRUKER). The samples for ESR spectroscopy measurement were by the method reported in the reference expect that the solvent was changed to chlorobenzene-acetonitrile mixed solvent [27].

*Raman:* The Raman spectra were measured by a Raman spectrometer (Renishaw) with a laser excitation wavelength of 532 nm. The solid-state samples for Raman spectroscopy measurement were prepared by the method reported in the reference expect that the solvent was changed to chlorobenzene-acetonitrile mixed solvent [27].

*Space charge limited current (SCLC):* The I-V curves of the samples used for the conductivity and SCLC measurements were obtained by a Keithley 2400 source meter in dark condition.

*Incident photon-electron conversion efficiency (IPCE):* The IPCE spectra were measured with a Crowntech QTest Station 1000 AD.

*Trap density of states (tDOS) and electrochemical impedance spectroscopy (EIS):* The tDOS of the PSCs was derived from angle frequency-dependent capacitance (C-F) data. The impedance spectroscopy and C-F curves were characterized using a Precision Impedance Analyzer 6500B Series of Wayne Kerr Electronics.

*Transient photovoltage (TPV) and transient photocurrent (TPC):* The TPV and TPC measurements were tested using the Keysight DSOX6004A oscilloscope and a nanosecond laser as the light source. The wavelength of light was 532 nm.

*Capacitance–voltage* (*C-V*)*:* The *C-V* data were obtained from the TH2829C impedance test.

## Results and Discussion

### Formation of 1,4-DITFB-TBP Complex

**Fig. 1:**
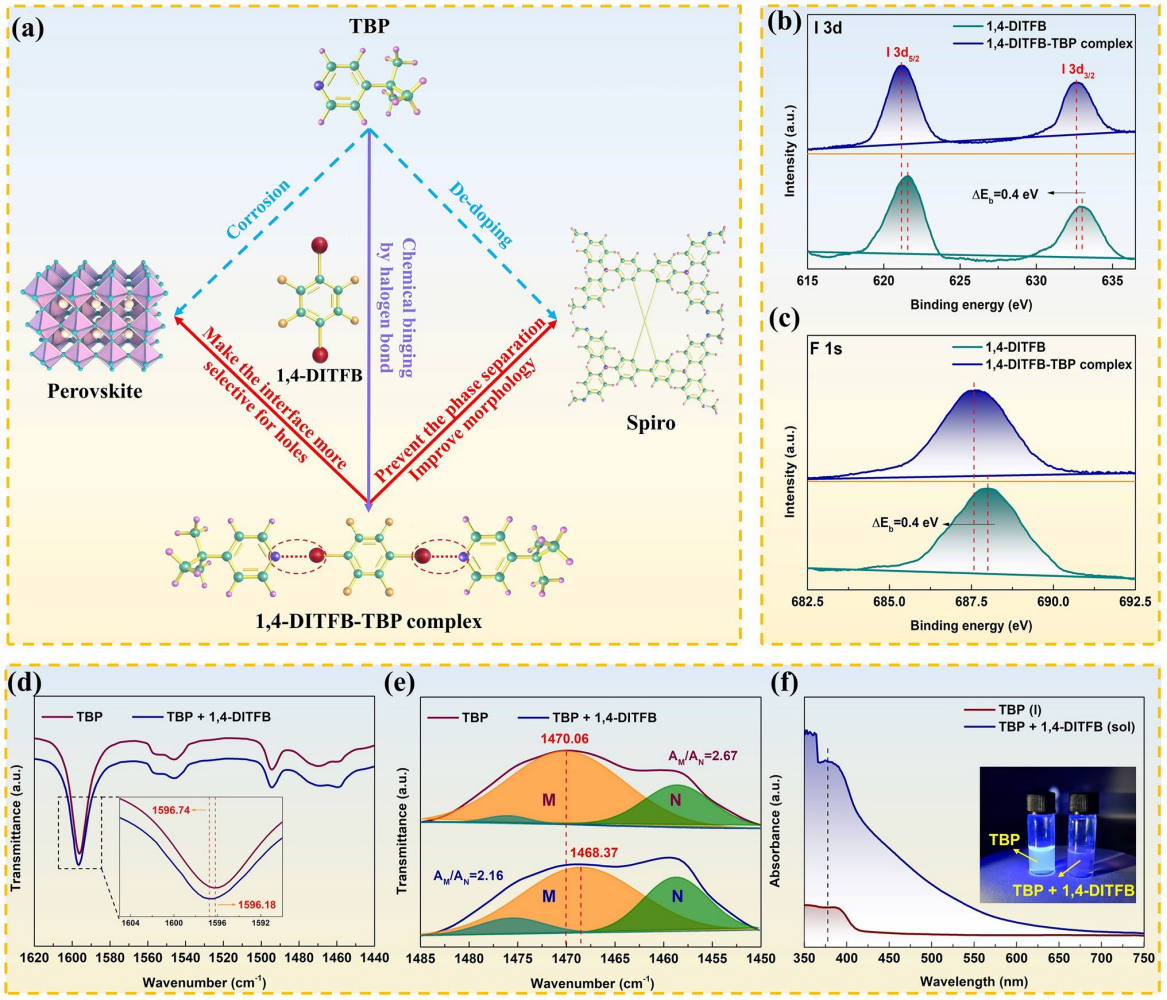
**a** Schematic illustration of the chemical binding process, and interactions between TBP, 1,4-DITFB-TBP complex, perovskite and spiro. (The red solid arrows show positive effects, while the blue dashed arrows show negative effects.) High-resolution XPS spectra depicting the **b** I 3*d* and **c** F 1* s* peaks of 1,4-DITFB and 1,4-DITFB-TBP complex. FTIR of pure TBP and 1,4-DITFB-TBP solution in the **d** 1440–1620 cm^−1^ and **e** 1450–1485 cm^−1^ range, and the inset is the partial magnification of the spectra. **f** Absorption spectra of pure TBP and 1,4-DITFB-TBP solution. Inset is the optical image of pure TBP and 1,4-DITFB-TBP solution under an UV lamp

As illustrated in Fig. 1a, TBP results in corrosive decomposition of perovskite and de-doping of spiro, which arises from the pyridine ring. Therefore, halogen bond is introduced to bind TBP and form 1,4-DITFB-TBP complex. The appropriate amount of 1,4-DITFB-TBP complex will not substantially affect the positive effects of TBP, which prevent the phase separation, improve morphology and make the interface more selective for hole. To verify the formation of this complex, XPS of pure 1,4-DIFTB and 1,4-DIFTB-TBP mixture was measured (Fig. 1b–c). The binding energy of I 3*d* shifts to the low binding energy region when adding TBP, and the same phenomenon is observed in the F 1 s XPS spectra, suggesting the increase in electronic state density around I and F atoms. Meanwhile, FTIR is displayed in Figs. 1d–e and S1. Formation of 1,4-DITFB-TBP complex is based on the lone electron pairs of N atoms because the peak of pyridine ring stretching around 1596 cm^−1^ shows a blue-shift, which will prevent the harmful reaction with PbI_2_ [24, 33]. The peak in the range of 1450–1485 cm^−1^ is the asymmetric vibrations of the tert-butyl group [33], which can be divided it into peak_M_ and peak_N_. It is observed that the peak_M_ position and the ratio of peak area *A*_M_/*A*_N_ change, demonstrating that there is indeed an interaction between 1,4-DITFB and TBP. The absorption spectra of pure TBP and 1,4-DITFB-TBP solution are displayed in Fig. 1f. An absorption peak appears at 377 nm for the 1,4-DITFB-TBP solution, which is different from the characteristic peaks of TBP and 1,4-DITFB [34], thereby proving the generation of 1,4-DITFB-TBP complex. Furthermore, TBP can luminesce under an ultraviolet (UV) lamp irradiation, while such phenomenon is almost invisible with the addition of 1,4-DITFB (Inset of Fig. 1f). The characterizations above qualitatively confirmed that 1,4-DITFB and TBP form the complex by halogen bonds. In subsequent studies, 1,4-DITFB-TBP complex (simply named as TBP complex) is pre-prepared and then added into spiro solution.

### Suppression of Perovskite Decomposition by TBP Complex

**Fig. 2:**
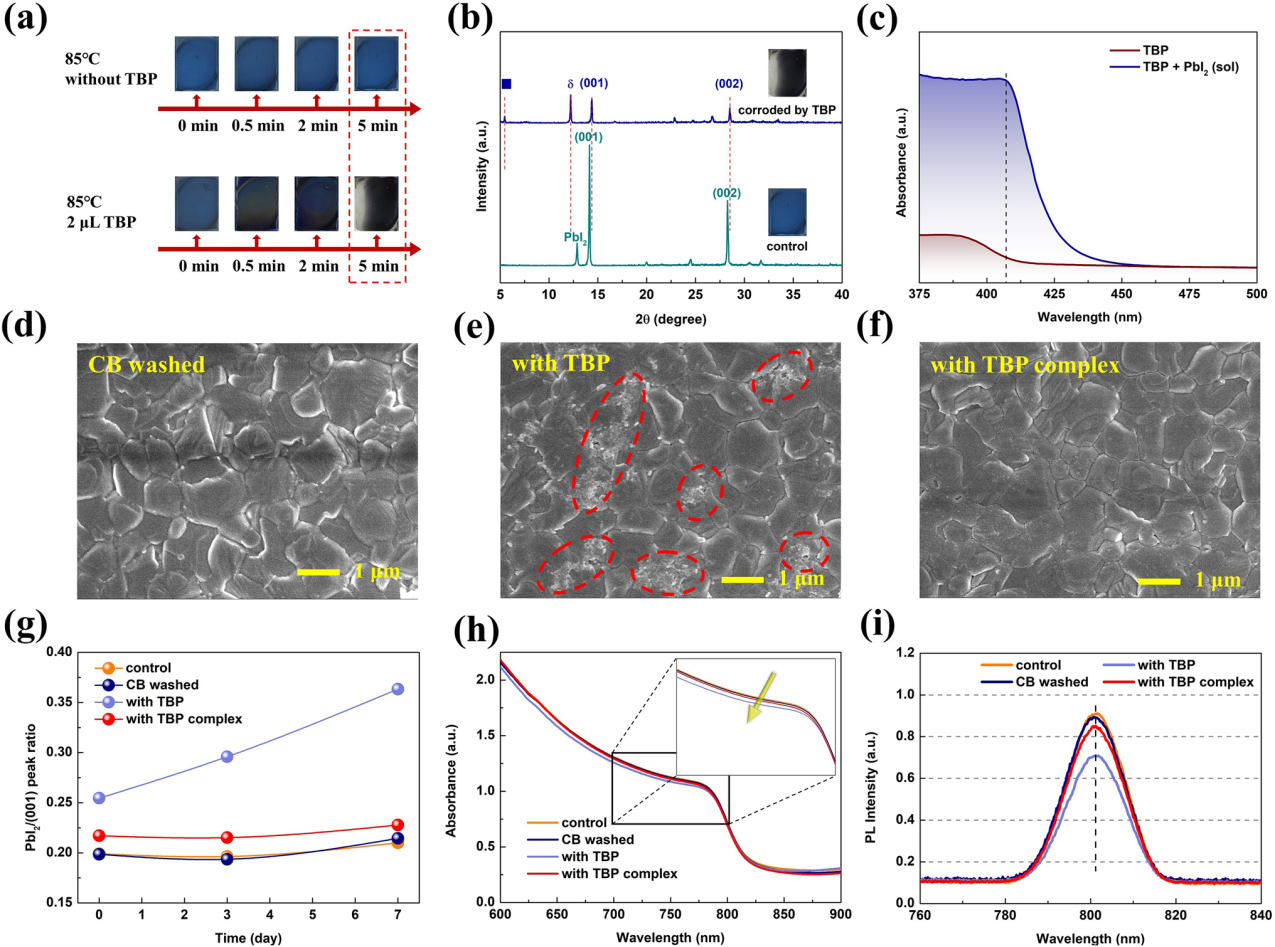
**a** Photographs of the evolution of FA_1-x_MA_x_PbI_3_ perovskite films at 85 °C without and with TBP. **b** XRD patterns of the perovskite film corroded by TBP and control film. **c** Absorption spectra of pure TBP and PbI_2_-TBP solution. The red dotted line is obtained by subtracting the absorption of pure TBP from the absorption of PbI_2_-TBP solution. SEM images of the **d** control film with CB washing, perovskite films once coated with spiro layer with **e** TBP and **f** TBP complex after 7 days of storage in N_2_. **g** PbI_2_/(001) peak ratio of the films as the function of time. Data are extracted from XRD measurement. Corresponding **h** absorption spectra and **i** steady-state PL spectra. Inset is the partial magnifications of the absorption spectra

Then, we explored the inhibition effect of TBP complex on perovskite decomposition derived from TBP. The detailed corrosion mechanism of TBP on FA_1-x_MA_x_PbI_3_ perovskite was obtained from the following phenomena and measurements. It can be found that FA_1-x_MA_x_PbI_3_ films were severely corroded by TBP when heating at 85 °C within 5 min compared with the control film at the same temperature (Figs. 2a and S2a). And XRD was carried out to demonstrate the change of perovskite crystal structure (Fig. 2b). The diffraction peaks at 14.14° and 28.26° of α phase perovskite are significantly decreased, while the peak of δ phase perovskite is greatly increased, suggesting that the standard perovskite crystal structure has been badly damaged [35, 36]. The slight shift of diffraction peaks of corroded film may arise from the lattice shrinkage. And PbI_2_ peak disappears accompanied by the formation of a new diffraction peak (marked with a blue square), indicating the formation of new species. The SEM and AFM measurements also demonstrate that the morphology of this film has been destroyed (Fig. S3). We also observed that perovskite film is seriously corroded at room temperature with 2 µL of TBP in Fig. S2b, indicating that PSCs will inevitably degenerate during device operation and storage even in the present of extremely few TBP in spiro layer. In order to prove TBP in HTL can destroy the perovskite film, SEM images of perovskite without and with spiro layer covering are performed after oxidation, and triple TBP is added into spiro solution to more clearly observe the change of perovskite morphology. Before measurements, perovskite layer covered with spiro layer was washed off twice with 50 μL chlorobenzene (CB) to remove spiro. By comparison, the control film is also washed by CB. In Fig. S4, the control film exhibits a uniform morphology with grain sizes of 0.5–2.5 μm, while many pits and small particles exist on the surface of perovskite film once covered by spiro, proving the corrosiveness of TBP. Meanwhile, the PSCs based on spiro with triple TBP have poor efficiency and stability (Fig. S5), while the device with triple TBP complex provides the improved efficiency and stability (Fig. S6), and detailed photovoltaic parameters are summarized in Table S1. We reasonably deduce that the devices with regular TBP still have potential risk to deteriorate with enough time and temperature.

The absorption spectra of pure TBP and PbI_2_-TBP solution are shown in Fig. 2c. With the addition of PbI_2_, an absorption peak appears at 407 nm and distinguishes from the characteristic peaks of TBP and PbI_2_ [37], proving the formation of new species, which can also explain the disappearance of PbI_2_ peak in Fig. 2b. Pure PbI_2_ powder and the product obtained by drying PbI_2_-TBP solution were further characterized by XPS. As illustrated in Fig. S7, the peaks of Pb 4*f* shift to a lower binding energy after TBP treatment, revealing that TBP interacts with the center Pb to form a complex [38]. Accordingly, the TBP-induced degradation mechanism can be concluded as the following reactions:

1 $$ {\text{FA}}_{{\text{1 - x}}} {\text{MA}}_{{\text{x}}} {\text{PbI}}_{{3}} {\text{(s) }} \leftrightarrow {\text{ FAI (s) + MAI (s) + PbI}}_{{2}} {\text{(s)}} $$

2 $$ {\text{PbI}}_{{2}} {\text{(s) + xTBP (s) }} \to {\text{ PbI}}_{{2}} {\text{ - TBP}}_{{\text{x}}} {\text{ (aq)}} $$

3 $$ {\text{PbI}}_{{2}} {\text{(s) + xTBP (s) }} \to { }\left[ {{\text{PbI}}_{{2}} \cdot {\text{xTBP}}} \right]{\text{ (s)}} $$

The formation of PbI_2_-TBP complex will further induce the perovskite decomposition according to Le Chatelier's principle. With this mechanism, a large number of defects generates on the perovskite surface, which is also the main route of ion migration [39]. The device degradation can be more severe even with advanced sealing techniques.

In view of the mechanism of perovskite degradation, PbI_2_ was added to the TBP solution of 1,4-DITFB until saturation, and no precipitation of 1,4-DIFTB was observed, manifesting that PbI_2_ could not destroy the chemical reaction between 1,4-DITFB and TBP. Subsequently, we performed a series of measurements on the perovskite films. Figure 2d–f provides the SEM images of the control film with CB washing, perovskite films once coated with spiro layer with TBP and with TBP complex after 7 days of storage in N_2_ (see details in Supporting Information). The films with TBP were seriously corroded (pinholes and small particles appear on the surface), while the perovskite film with TBP complex was essentially unchanged and remained uniform. Even if the perovskite films with spiro layer were stored for 3 day or have just completed oxidation, the obvious morphology damage can be observed (Fig. S8). AFM images are consistent with the SEM results (Fig. S9). The root-mean-square (RMS) roughness of the film with TBP after 7 days is 53.1 nm, much higher than the other three samples.

XRD was utilized to observe the relative changes of characteristic peaks of perovskite and PbI_2_. As displayed in Fig. S10, all samples exhibit diffraction peaks of (001), (002), and (111) crystal planes of FA_1-x_MA_x_PbI_3_ perovskite, accompanied by the PbI_2_ peak [35]. The PbI_2_/(001) peak ratio as the function of time is shown in Fig. 2g. The reduced ratio for the films with TBP complex demonstrates that the corrosive decomposition of perovskites is mitigated. For corresponding absorption spectra in Figs. 2h and S11, the light absorbance of the perovskite film once covered with spiro doping with TBP complex is higher, and the absorption intensity is relatively stable. The similar trends can be concluded from the difference in PL peak intensity (Figs. 2i and S12). More intuitively, the normalized PL intensity versus time for the samples is extracted into Fig. S12c. The measurements and characterization above have fully proved that TBP can induce perovskite decomposition and destroy crystal quality. The chemical binding from halogen bonds inhibits the reaction of TBP and PbI_2_, which successfully mitigates the corrosion effect. Furthermore, we employed AFM and SEM to observe the role of TBP complex on the morphology and microstructure of the spiro film. The spiro film with TBP shows many round-shaped bulges with the RMS roughness of 1.99 nm (Fig. S13b). With TBP complex, the film becomes smooth with the RMS roughness decreased to 1.14 nm (Fig. S13c). It can be attributed that TBP complex slows down the evaporation of TBP, which is a suitable morphology controller as previously described [40, 41]. The SEM images in Fig. S14 indicate that the spiro with TBP complex presents the same surface morphology as the one with TBP.

### Suppression of Spiro De-doping by TBP Complex

**Fig. 3:**
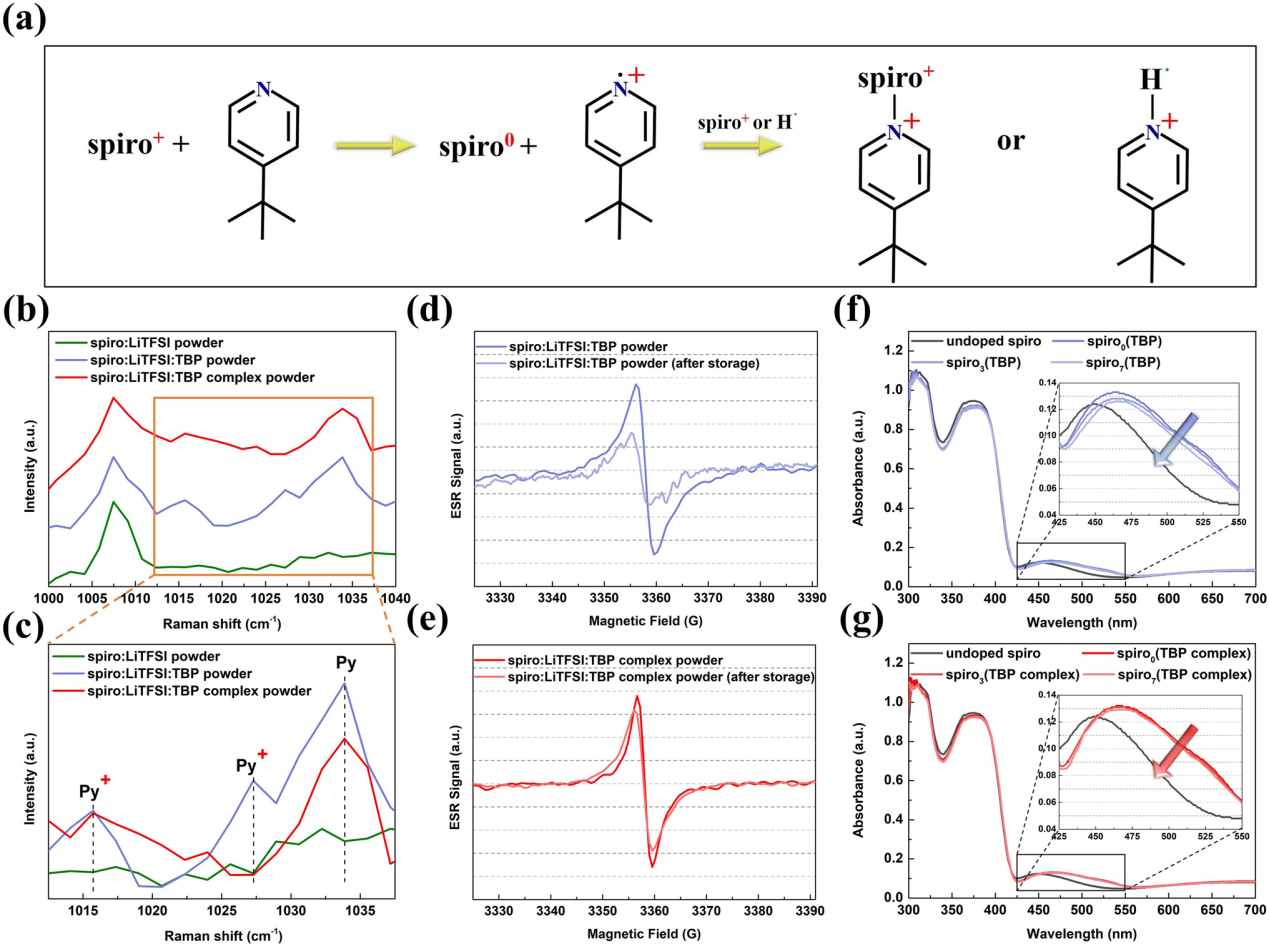
**a** Reaction mechanism for TBP-induced spiro de-doping. **b** Raman spectra of the different spiro-based powders in the 1000–1040 cm^−1^ range. **c** Magnification of the spectra in the 1012.5–1037.5 cm^−1^. ESR spectroscopy of the different spiro-based powders with **d** TBP and **e** TBP complex. Absorption spectra of different spiro films with **f** TBP and **g** TBP complex. Insets are the partial magnifications of the spectra

Furthermore, the TBP complex is also expected to suppress the TBP-induced spiro de-doping. Figure 3a gives the chemical reactions in spiro layer during oxidation and storage [27]. Spiro^+^ molecules can increase the HTL conductivity, but the present of TBP reduces its content, which is detrimental to PSCs with FF loss and unexpected charge recombination. We conducted Raman spectroscopy measurement on the different spiro-based samples in the solid state in Fig. S15. The shift of the peak around 732 cm^−1^ toward higher energy confirms that TBP does affect spiro and tends to its amorphous form [27]. The variation of peaks in the range of 1000–1040 cm^−1^ is particularly concerned (Fig. 3b). A peak centered at 1007.5 cm^−1^ is visible for all three samples, which may be the evidence for the mixing of spiro and LiTFSI. The Raman peaks around 1015 and 1027 cm^−1^ were assigned to pyridinium cation species (Py^+^), while the peak around 1034 cm^−1^ originated from neutral pyridine species (Py), which is labeled in Fig. 3c [42]. Obviously, the Py^+^ peak disappears for the spiro:LiTFSI:TBP complex powder, suggesting that 1,4-DITFB reduces the generation of Py^+^. Considering the enhanced molecular diffusion in solution state, the solution samples with the same composition as the powder were also characterized. Interestingly, the solution samples have similar Raman peaks as the powder samples, but the signals of Py^+^ and Py become intensive, which is beneficial for our analysis (Fig. S16a–b). The peak area ratio of Py^+^ and Py for spiro:LiTFSI:TBP sample is 0.2619, but the ratio for spiro:LiTFSI:TBP complex sample decreases to 0.1970 (Fig. S16c–d). This is the same as the Raman data of the powder samples.

ESR spectroscopy is an effective tool to detect spiro^+^ radicals. Lamberti et al. used ESR spectroscopy to explore the de-doping process of spiro in detail [27]. In Fig. S17, the pure spiro sample displays no obvious radical signals. In contrast, the spiro:LiTFSI sample presents strong signals to evidence the existence of spiro^+^ radicals. The addition of TBP significantly reduces the ESR signal intensity, while the sample with TBP complex gives a stronger signal than the sample with pure TBP. Then, we stored these two samples in dark and N_2_ conditions for 7 days. The sample with TBP shows an obvious intensity decrease and shape change (Fig. 3d), which is similar to previous report [27]. The TBP complex can suppress this phenomenon, as observed by the mitigated signal attenuation in Fig. 3e. Absorption spectra were used to qualitatively analyze the variation of spiro^+^ content in spiro films. As exhibited in Fig. 3f–g, the absorption peaks around 523 nm have been assigned to the spiro^+^ [43]. Tracing the evolution of the peak intensity allows us to observe the increase or decrease of spiro^+^. The spiro-based films were measured for three time periods and represented by subscripts 0, 3, and 7, respectively. The spiro^+^ peak of the sample with TBP slightly decreased after storage for 3 and 7 days. In comparison, the peaks of the three samples with TBP complex reveal a negligible difference. The samples in solution state were measured and are illustrated in Fig. S18. Due to the accelerated intermolecular interaction and possible solvent volatilization, the absorption peak of spiro^+^ decreases. Similarly, the absorbance of spiro(TBP) is much lower than that of the sample with TBP complex. Subsequently, we measured the electrical conductivity (σ_0_) of the spiro films with the device structure of ITO/spiro/Ag (Figs. S19 and S20) [44]. The detailed values are listed in Table S2. The σ_0_ of the spiro_0_(TBP complex) film is 1.75 × 10^–5^ s cm^−1^, slightly higher than the spiro_0_(TBP) film (1.68 × 10^–5^ s cm^−1^). After 7 days of storage, the loss of σ_0_ is about 13.69% (1.45 × 10^–5^ s cm^−1^), but the spiro(TBP complex) film only loses 4.57% of the initial σ_0_ (1.67 × 10^–5^ s cm^−1^). Figure S21 clarifies that the spiro_0_(TBP) film and spiro_0_(TBP complex) film have very similar band structures. The measurements and analysis prove that the complex reduces the TBP-induced reduction of spiro^+^ without impairing the film electrical properties, consequently enabling the spiro HTL with better stability.

### Performance and Stability of PSCs

**Fig. 4:**
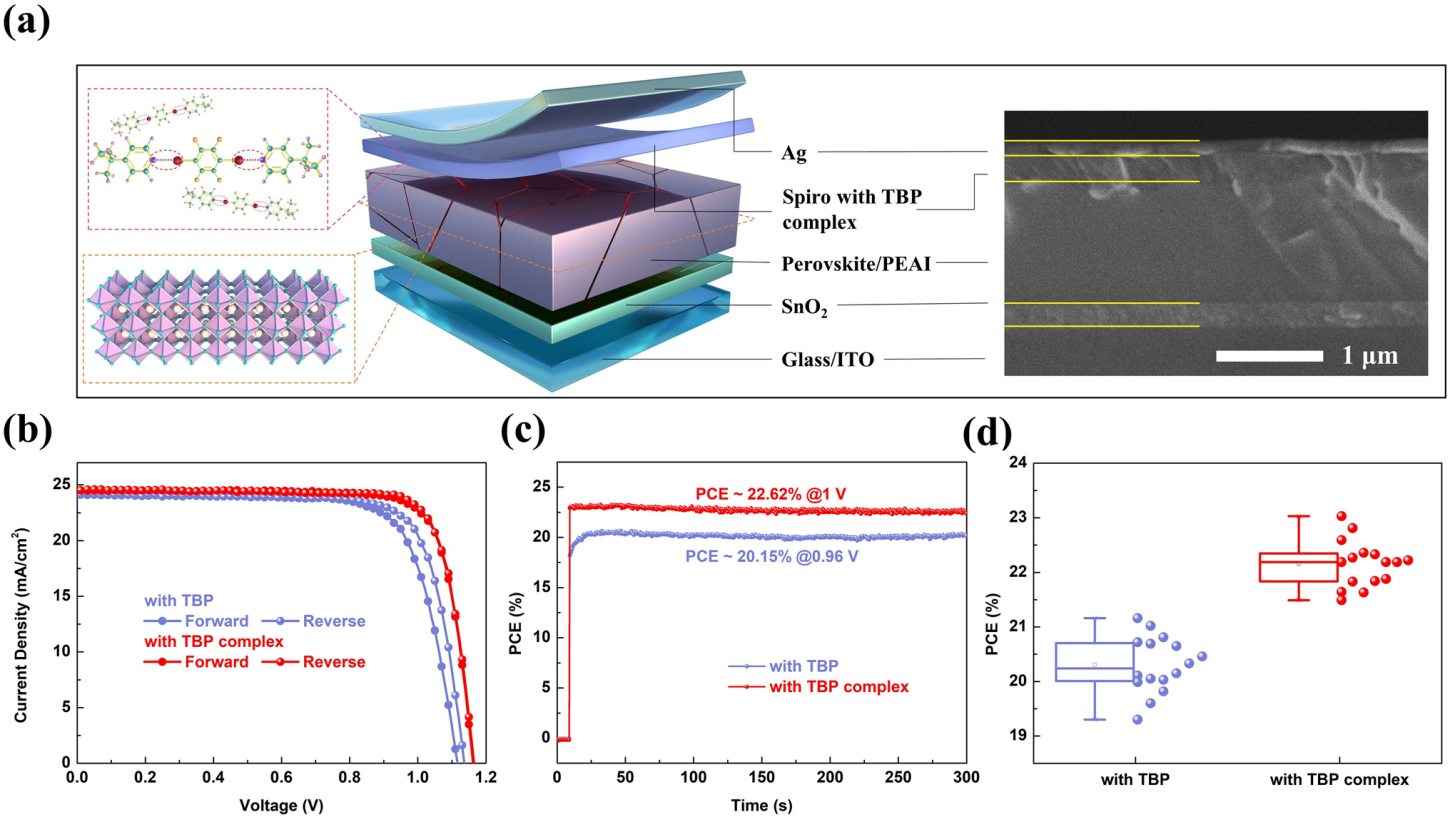
**a** Structure diagram and corresponding cross-sectional SEM image of fabricated PSCs. **b** *J-V* curves of the devices with TBP and TBP complex measured by forward and reverse scans. **c** Stabilized PCEs. **d** Distribution of the PCE of the devices with TBP and TBP complex (16 individual cells are included)

With improved stability of the perovskite layer and spiro HTL, the device performance was subsequently investigated. Device structure is displayed in Fig. 4a, as well as the corresponding cross-sectional SEM image. The concentration of 1,4-DITFB was optimized, and the most suitable concentration was determined to be 2 wt%. Figure S22 and Table S3 show the champion *J-V* curves and photovoltaic parameters of these PSCs. As summarized in Fig. 4b and Table S4, the device with TBP delivers a high PCE of 21.16%. With TBP complex, the device provides a champion PCE of 23.03%, with a *V*_oc_ of 1.165 V, a *J*_sc_ of 24.50 mA cm^−2^, and a FF of 80.70%. In addition, the hysteresis index (HI) reduces from 4.58 to 1.09% [45]. The enhanced PCE and suppressed hysteresis mainly result from the high quality of the perovskite and the reduced defects in the device, as well as the slight improvement in spiro layer. And *J*_sc_ is consistent with the integrated *J*_sc_ from IPCE spectra in Fig. S23. In Figs. 4c and S24, we can find that the devices with TBP complex demonstrate faster response, indicating more efficient charge extraction than the devices with TBP. Figures 4d and S25 give the distribution of the photovoltaic parameters of 16 individual cells employing the spiro with different contents of 1,4-DITFB.

**Fig. 5:**
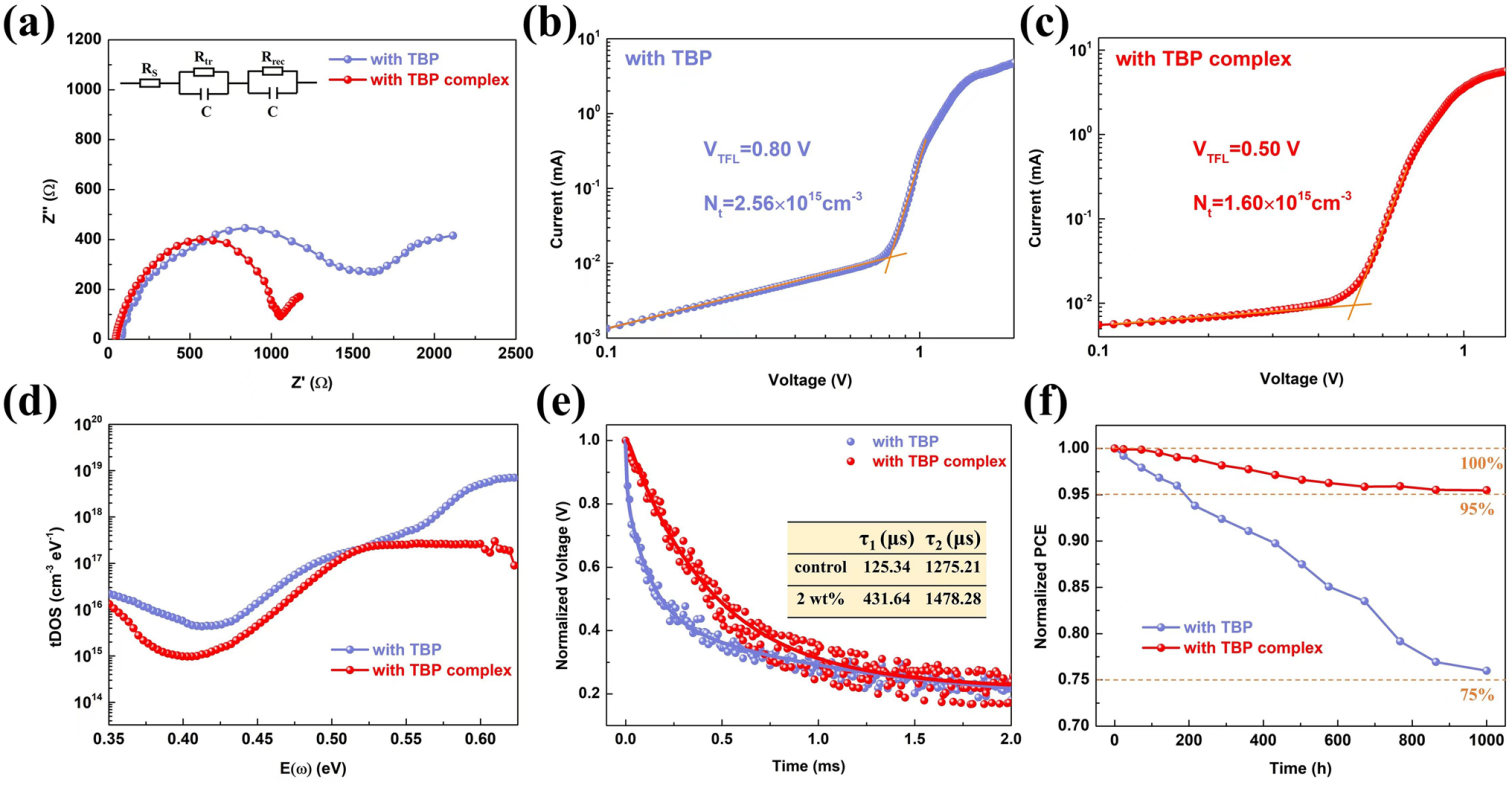
**a** Nyquist plots of the devices with TBP and TBP complex measured under 0.8 V bias voltage, and the inset is the equivalent circuit. **b, c** are the *I-V* curves of the hole-only devices with the structure of ITO/PEDOT:PSS/FA_1-x_MA_x_PbI_3_/spiro with TBP or TBP complex/Ag. **d** tDOS for the devices with TBP and TBP complex. **e** TPV based on the devices with TBP and TBP complex. **f** PCE evolution of the devices with TBP and TBP complex without encapsulation in N_2_ atmosphere at room temperature

EIS was commonly performed to study the charge transport and carrier recombination in PSCs [46]. The Nyquist plots measured under illumination and different bias voltages consist of two separate arcs, as exhibited in Figs. 5a and S26. According to the equivalent circuit, it can be seen that the series resistance (*R*_S_) and transfer resistance (*R*_tr_) of the device with TBP complex are both lower than those of the device with TBP, while the recombination resistance (*R*_rec_) is the opposite. The results at all bias voltages follow the above analysis, clarifying that enhanced hole transport efficiency and reduced carrier recombination are achieved. Halogen bonds can protect perovskites from corrosion decomposition, so as to reduce the formation of defects. Then, the hole-only device with ITO/PEDOT: PSS/FA_1-x_MA_x_PbI_3_/spiro/Ag geometry was constructed and the space charge limited current (SCLC) method was used to calculate the trap density (*N*_t_) of perovskite films [47]. With the complex formed through halogen bonds, the N_t_ of the perovskite film decreased from 2.56 × 10^15^ to 1.60 × 10^15^ cm^−3^ (Fig. 5b–c). The thermal admittance spectroscopy measurement was also conducted to analyze the tDOS of the device [48]. As presented in Fig. 5d, the tDOS of the device with TBP complex is reduced compared to the device with TBP. We measured TPV and TPC, and the corresponding curves are presented in Figs. 5e and S27. The device with TBP complex has a higher lifetime for photovoltage and a lower lifetime for photocurrent, revealing the suppressed non-radiative recombination and efficient charge transfer [49]. The curves of 1/C^2^-V were plotted to estimate the built-in voltages in Fig. S28. The device with TBP complex has a higher built-in voltage, and therefore, the capacity to separate electrons and holes is better, which is reflected in *V*_oc_ [50]. Importantly, the PSCs with TBP complex exhibit enhanced stability. In N_2_ atmosphere and room temperature, the device with TBP maintains about 75% of its initial PCE, while the PCE only reduces by 5% for the device with TBP complex after 1000 h storage (Figs. 5f and S29). Finally, we tested the humidity stability of the unencapsulated devices. With 40 ± 5% RH at room temperature, 32% and 10% of their initial PCE are lost for the devices with TBP and with TBP complex after 500 h, respectively (Fig. S30). The stability data at 60 ± 5% RH and about 85% RH were also measured and are shown in Fig. S31. Compared with the PSCs with TBP, the PSCs with TBP complex exhibit more outstanding stability (89% vs. 65% at 60 ± 5% RH and 72% vs. 41% at about 85% RH after 240 h).

## Conclusion

In summary, we developed a chemical binding strategy via halogen bonds to suppress the TBP-induced instability in PSCs. 1,4-DIFTB was applied to interact with the N atom in TBP. After the formation of 1,4-DITFB-TBP complex, the decomposition of perovskites is significantly alleviated, and the de-doping of spiro is suppressed. The perovskite film with reduced defects and improved crystal quality is achieved. Meanwhile, the spiro HTL well maintained its excellent conductivity, and all of which have been comprehensively proved. As a result, the integrated device with TBP complex achieves a champion PCE of 23.03%. Moreover, the unencapsulated device based on the spiro with TBP complex retains about 95% of its initial PCE after stored for 1000 h in the condition of N_2_ and room temperature, as well as excellent humidity stability. This work exemplifies the non-innocent role of TBP as the classic dopant for spiro HTL and raises the possibility for the application of TBP in highly stable PSCs, pushing the step closer to commercialization.

## Supplementary Information

Below is the link to the electronic supplementary material.

Supplementary file1 (PDF 2197 kb)
