# Supplementary material for: Overcoming Perovskite Corrosion and De-Doping Through Chemical Binding of Halogen Bonds Toward Efficient and Stable Perovskite Solar Cells
Source: Nanomicro Lett. 2022 Aug 23;14:175. doi: 10.1007/s40820-022-00916-3 (PMC9399337; doi:10.1007/s40820-022-00916-3)
Supplement: Supplementary file 1 — Supplementary file1 (PDF 2197 kb) [file 40820_2022_916_MOESM1_ESM.pdf]

Supporting Information for

Overcoming Perovskite Corrosion and De-Doping through Chemical Binding of Halogen Bonds towards Efficient and Stable Perovskite Solar Cells

Guanhua Ren[1], Wenbin Han[1], Qiang Zhang[1], Zhuowei Li[1], Yanyu Deng[1], Chunyu Liu[1, *], and Wenbin Guo[1, *]

[1]State Key Laboratory of Integrated Optoelectronics, College of Electronic Science and Engineering, Jilin University, 2699 Qianjin Street, Changchun 130012, People's Republic of China

*Corresponding authors. E-mail: Dr. chunyu_liu@jlu.edu.cn (C. Y. Liu), guowb@jlu.edu.cn (W. Guo)

Supplementary Figures and Tables

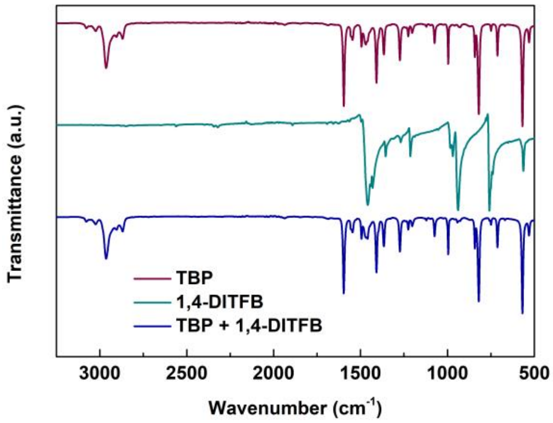

Fig. S1 Full range FTIR of TBP, 1,4-DITFB and 1,4-DITFB-TBP solution

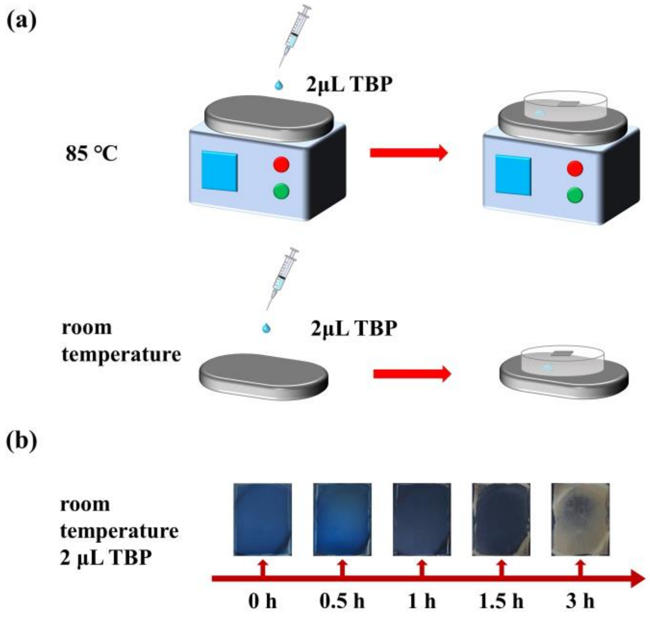

Fig. S2 a Schematic diagram depicting the evolution of $FA_{1-x}MA_xPbI_3$ perovskite films under different treatment conditions and times. **b** Photographs of the evolution of $FA_{1-x}MA_xPbI_3$ perovskite films at room temperature with 2 μL TBP

At high temperature: 2 μL TBPwas dropped on a hot stage at 85 °C, then a glass dish with the perovskite film on the top was immediately buckled on the TBP. Here 85 °C was chosen because the local temperature can reach this temperature during device operation [S1]. The control sample was treated in the similar way, but without TBP.

At room temperature: 2 μL TBPwas dropped on a metal substrate at room temperature, and the glass dish with the perovskite film on the top was immediately buckled on the TBP.

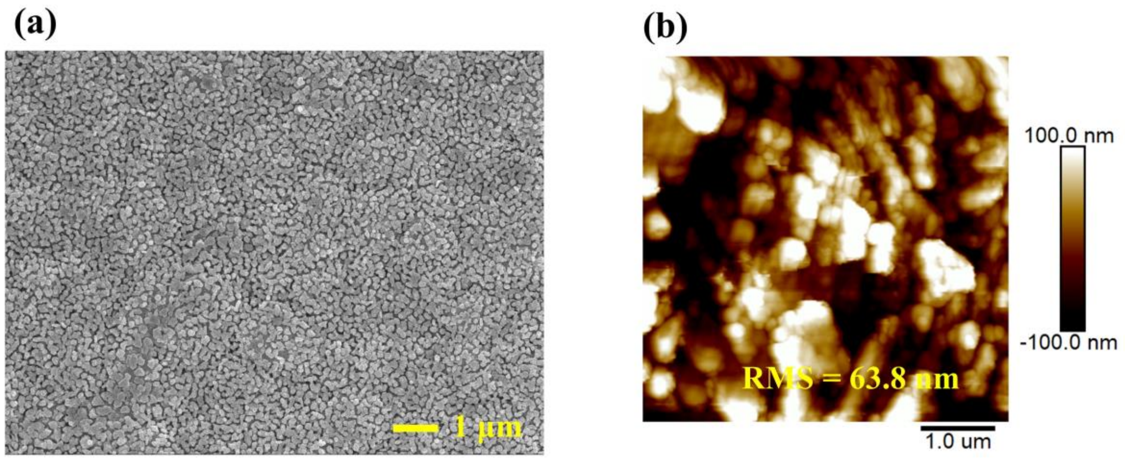

Fig. S3 a SEM and **b** AFM images of the perovskite film corroded by TBP

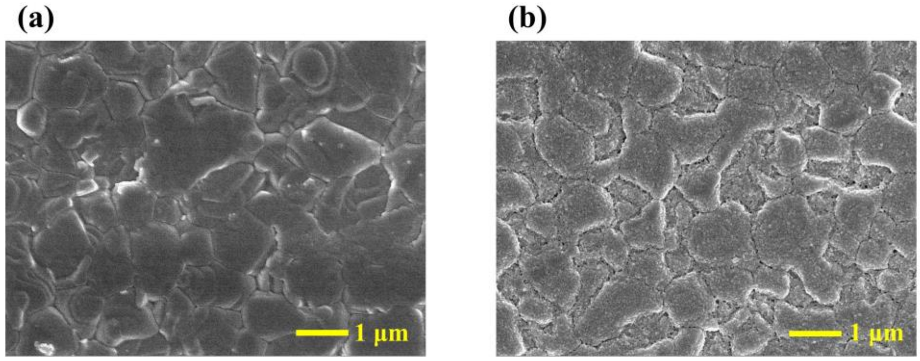

Fig. S4 SEM images of perovskite layers once **a** without and **b** with spiro spin-coating

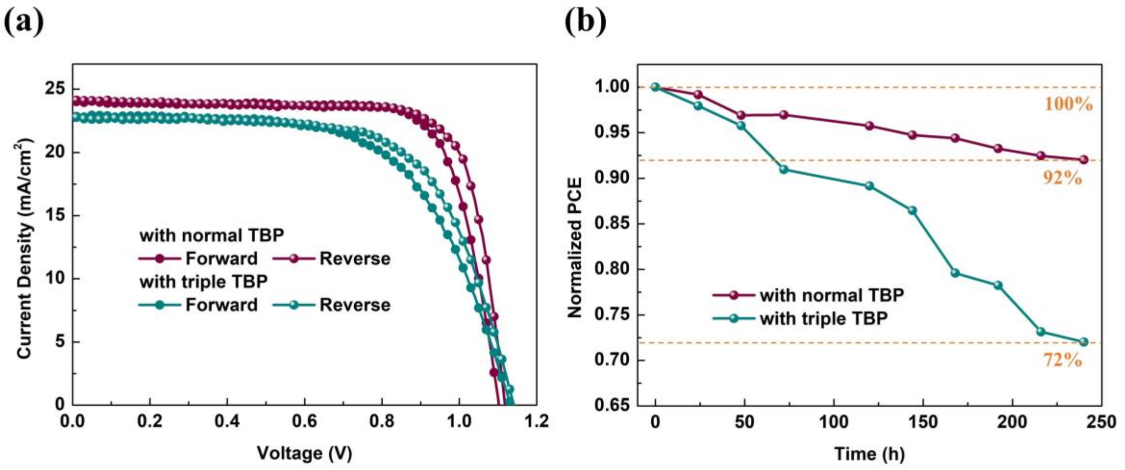

Fig. S5 a J-V curves of the devices with normal and triple TBP measured by forward and reverse scans. **b** PCE evolution of the unencapsulated devices with normal and triple TBP content in N_2 atmosphere at room temperature

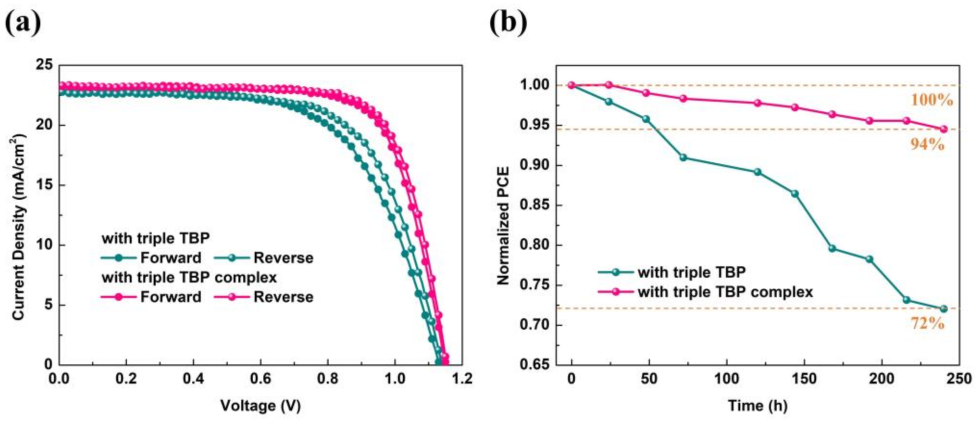

Fig. S6 a J-V curves of the devices with triple TBP and triple TBP complex measured by forward and reverse scans. **b** PCE evolution of the unencapsulated devices with triple TBP and triple TBP complex in N_2 atmosphere at room temperature

Although the TBP complex improves the performance and stability of the PSCs, the efficiency of the device with triple TBP is lower than that of the device with normal TBP because the three-fold usage is too high. In terms of the stability, the former is slightly better than that of latter.

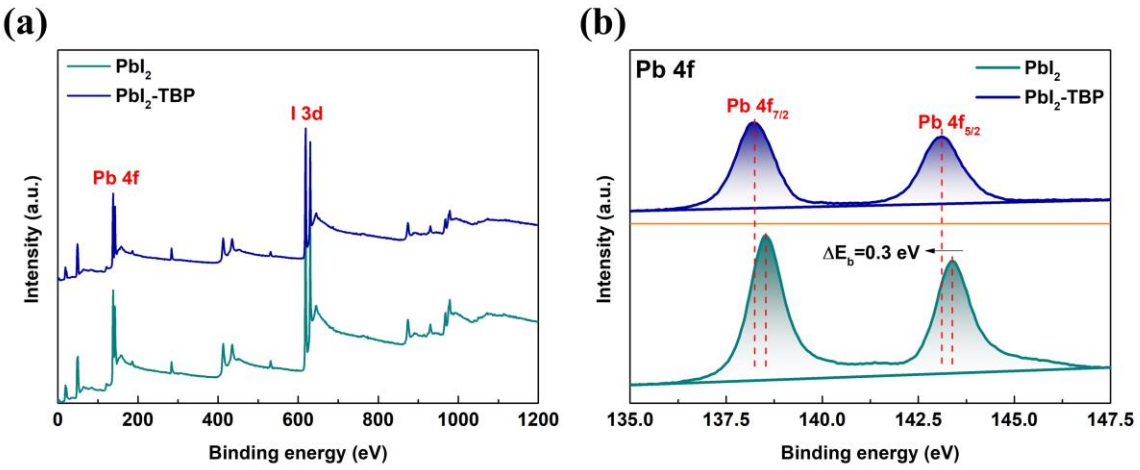

Fig. S7 a XPS survey of the PbI_2 and PbI_2-TBP. **b** High-resolution XPS spectra depicting the Pb 4f peaks of PbI_2 and PbI_2-TBP

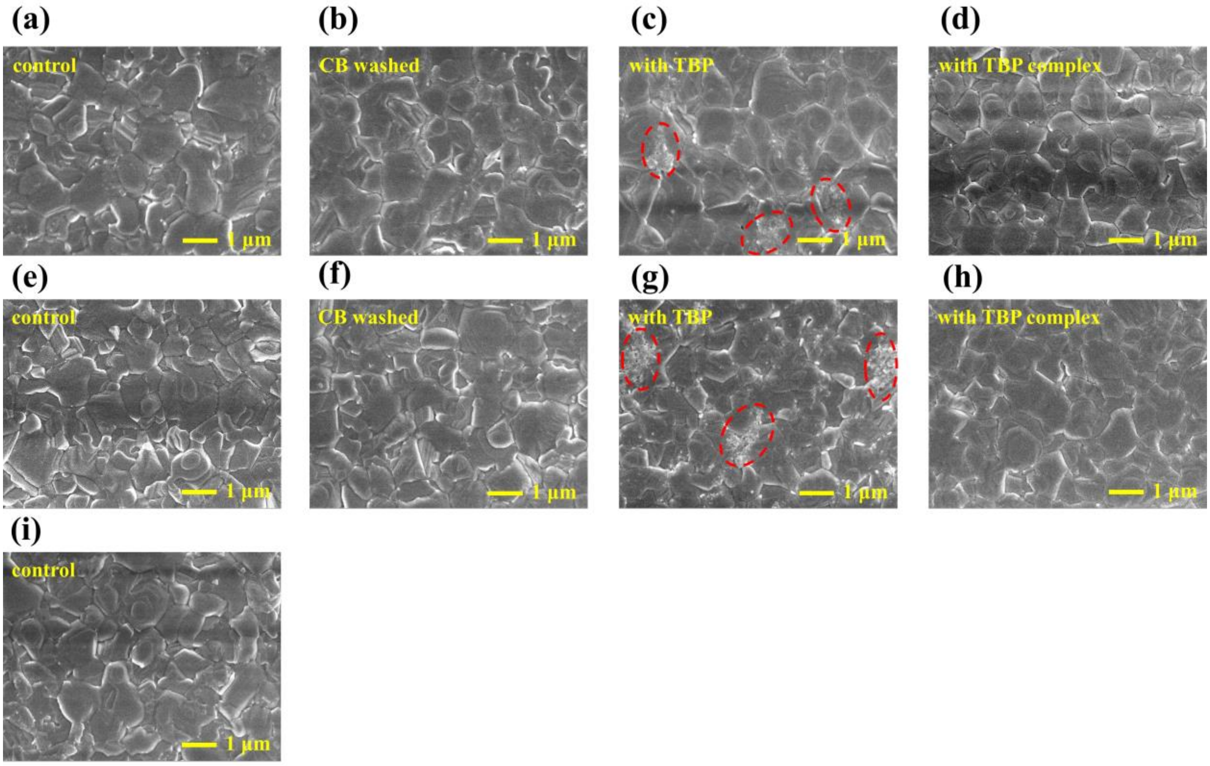

Fig. S8 SEM images of the control film **a** without and **b** with CB washing, perovskite films once coated with spiro layer with **c** TBP and **d** TBP complex just after oxidation. SEM images of the control film **e** without and **f** with CB washing, perovskite films once coated with spiro layer with **g** TBP and **h** TBP complex after 3 days of storage in N_2. **i** SEM image of the control film after 7 days of storage in N_2

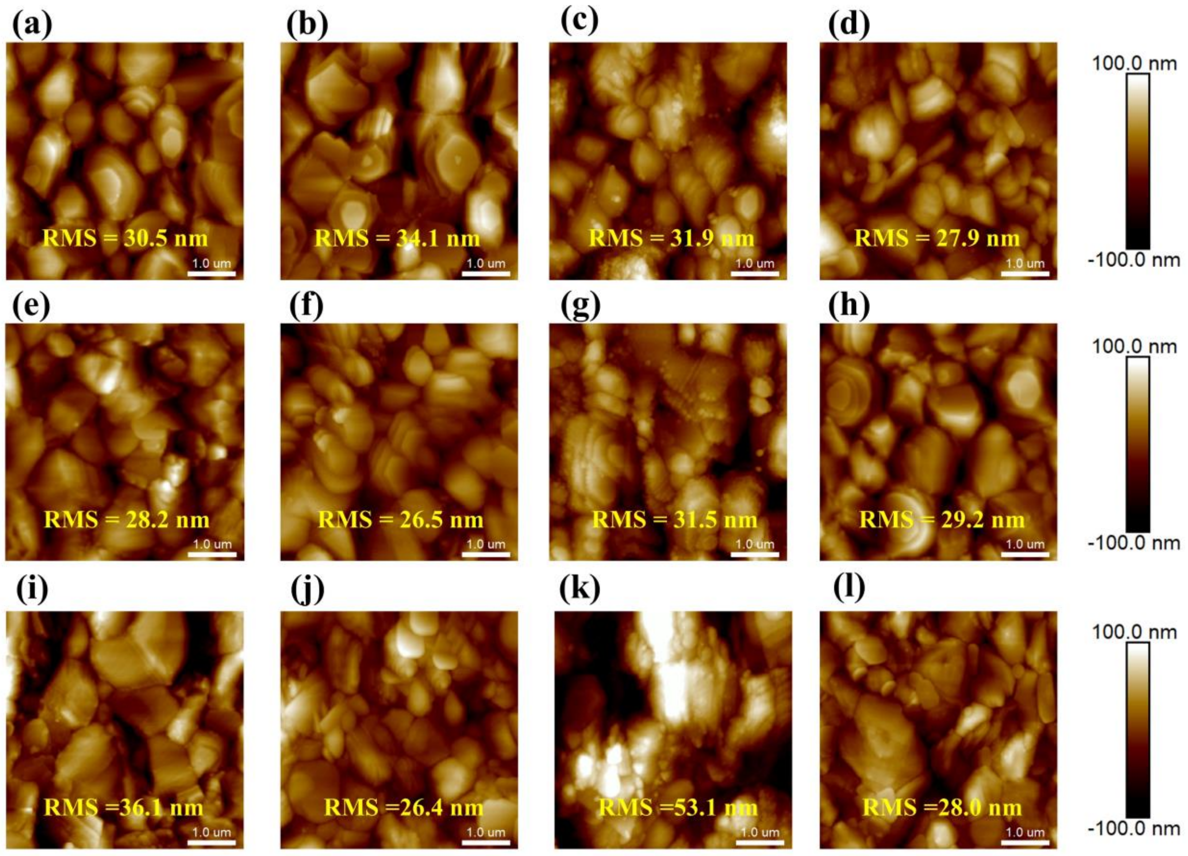

Fig. S9 AFM images of the control film **a** without and **b** with CB washing, perovskite films once coated with spiro layer with **c** TBP and **d** TBP complex just after oxidation. AFM images of the control film **e** without and **f** with CB washing, perovskite films once coated with spiro layer with **g** TBP and **h** TBP complex after 3 days of storage in N_2. AFM images of the control film **i** without and **j** with CB washing, perovskite films once coated with spiro layer with **k** TBP and **l** TBP complex after 7 days of storage in N_2

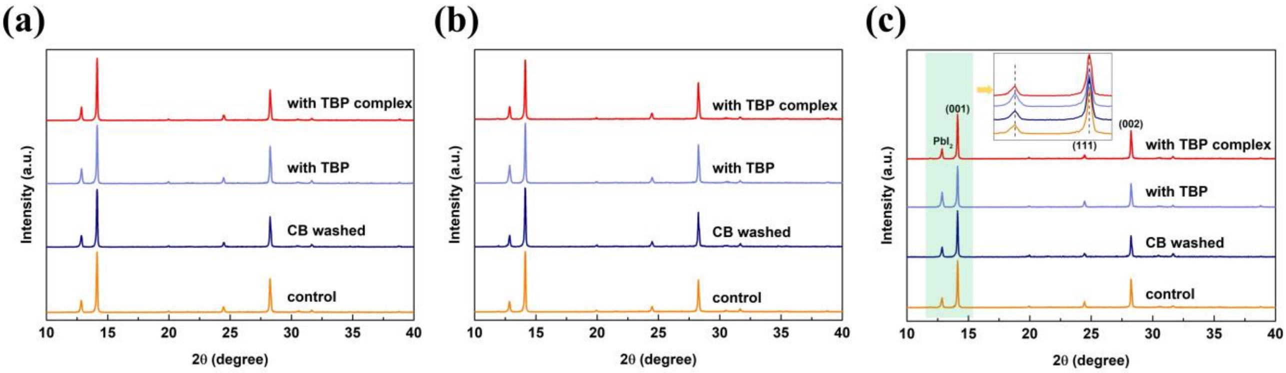

Fig. S10 XRD patterns of the samples **a** just after oxidation, **b** stored in N_2 for 3 days and **c** stored in N_2 for 7 days, including the control film without and with CB washing, perovskite films once coated with spiro layer with TBP and TBP complex. The inset is the partial magnifications of the XRD spectra

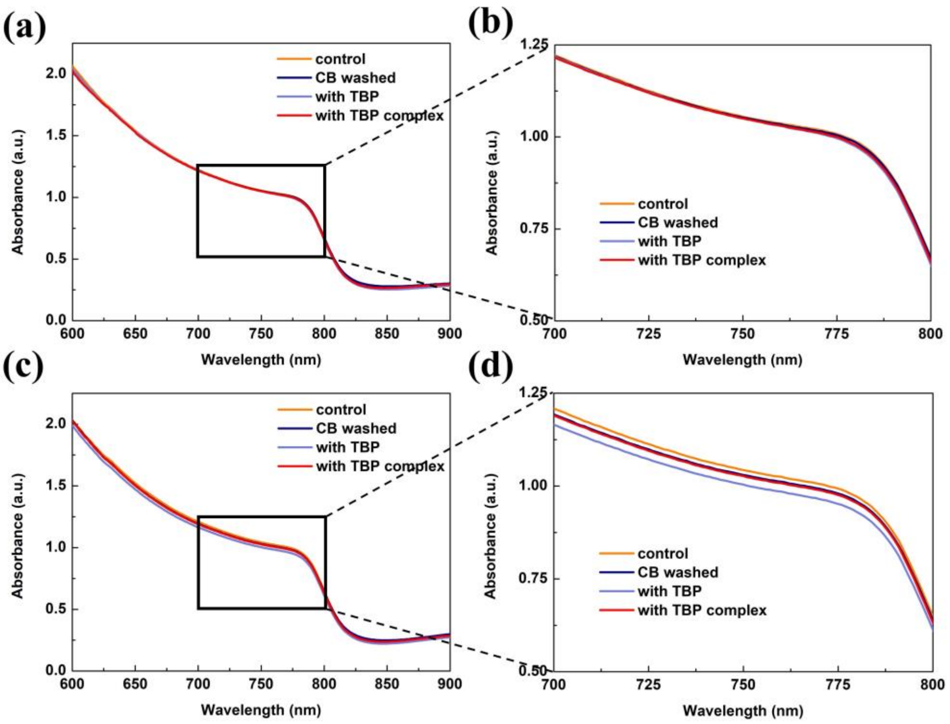

Fig. S11 Absorption spectra of the samples **a** just after oxidation, **c** stored in N_2 for 3 days, including the control film without and with CB washing, perovskite films once coated with spiro layer with TBP and TBP complex. **b** and **d** are the partial magnifications of the spectra

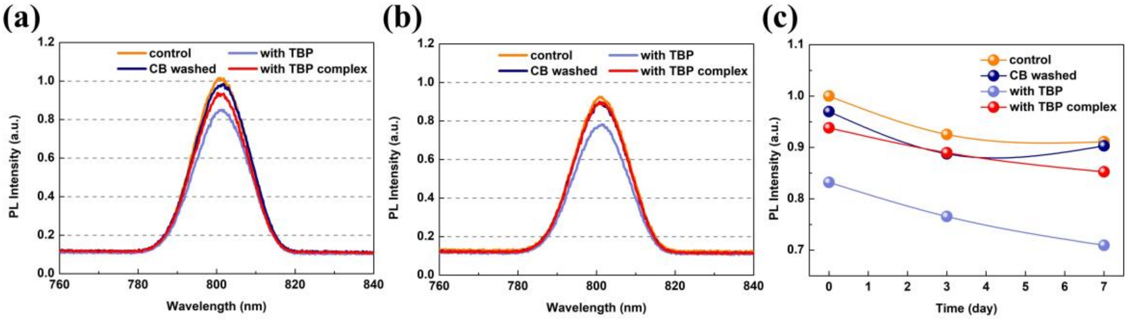

Fig. S12 Steady-state PL spectra of the samples **a** just after oxidation, **b** stored in N_2 for 3 days, including the control film without and with CB washing, perovskite films once coated with spiro layer with TBP and TBP complex. **c** PL intensity of these samples as the function of time

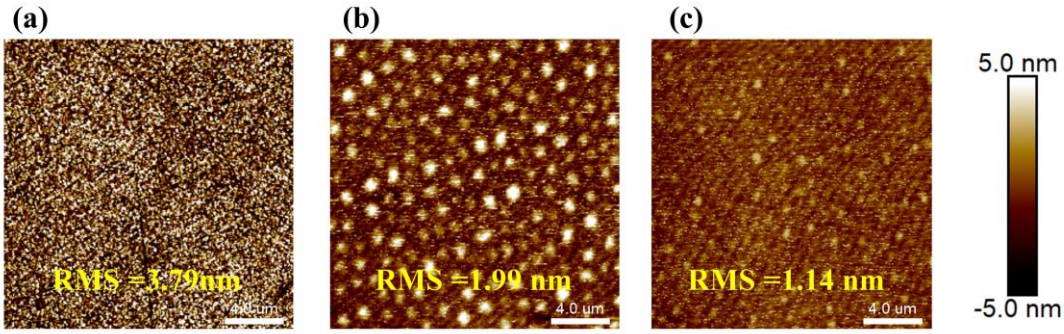

Fig. S13 AFM images of the **a** ITO substrate, spiro films with **b** TBP and **c** TBP complex

The RMS of the ITO substrate is 3.79 nm. The spiro films with TBP or TBP complex were spin-coated on the ITO substrate directly because the RMS of the perovskite film is high.

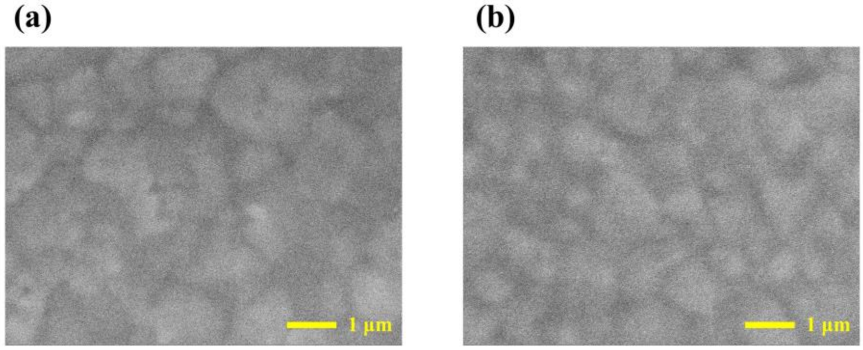

Fig. S14 SEM images of the spiro film with **a** TBP and **b** TBP complex

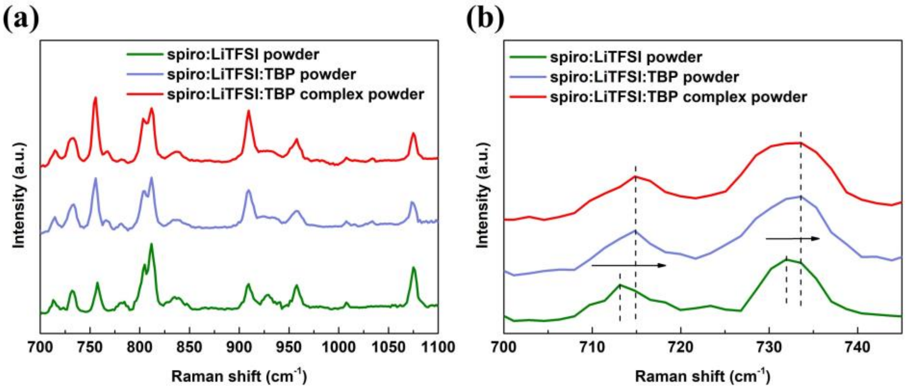

Fig. S15 Raman spectra of the different spiro-based powders in the **a** full range and **b** 700-745 cm^{-1} range. The peak around 732 cm^{-1} is assigned to the a1 fundamental mode of fluorenes, whose shift represents the change of lattice parameters [S2]. The analysis for the peaks around 715 cm^{-1} is difficult owing to the strong signal of TBP at this position [S3].

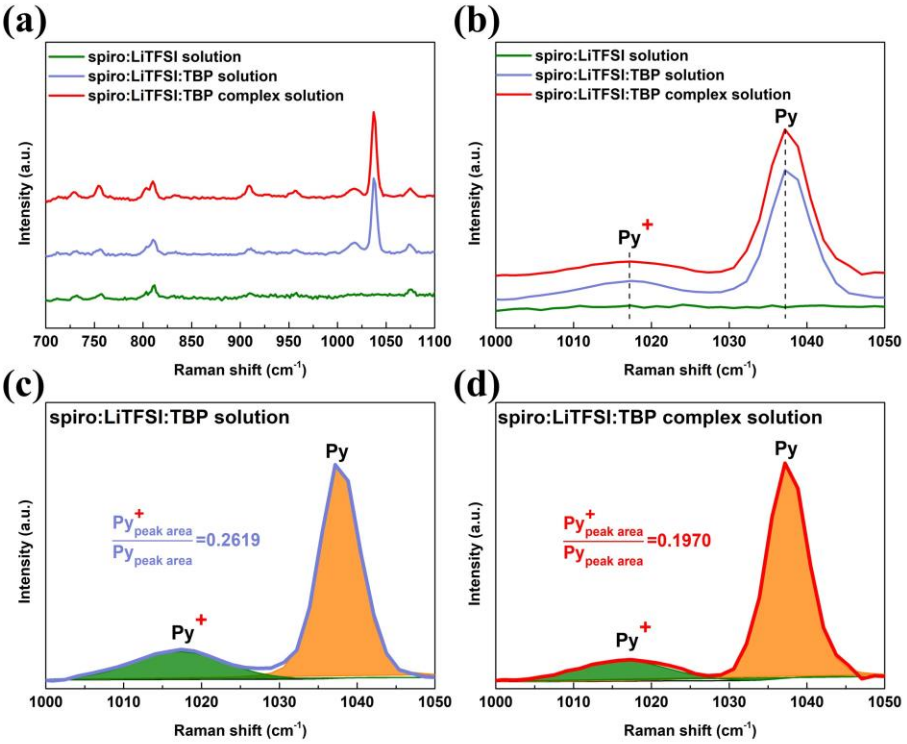

Fig. S16 Raman spectra of the different spiro-based solutions in the **a** full range and **b** 1000-1050 cm^{-1} range. The peak area ratio of Py^+ and Py for **c** spiro:LiTFSI:TBP solution and **d** spiro:LiTFSI:TBP complex solution

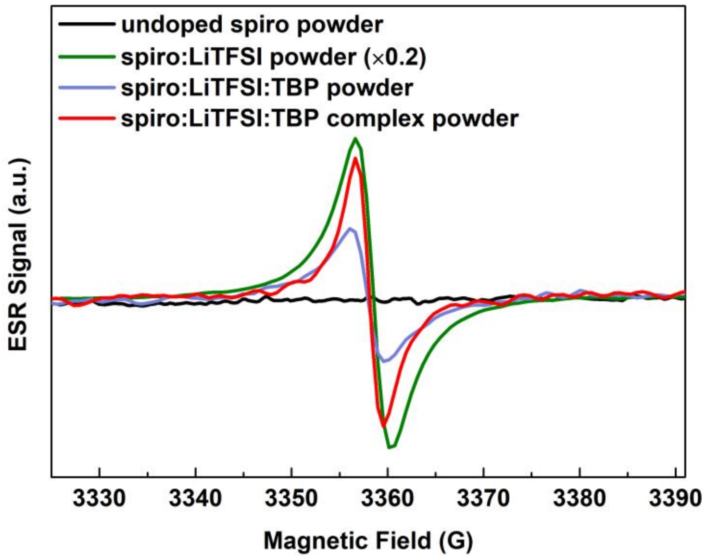

Fig. S17 ESR spectroscopy of the different spiro-based powders. The spiro:LiTFSI signal is reduced by a factor of 5 to improve data visualization

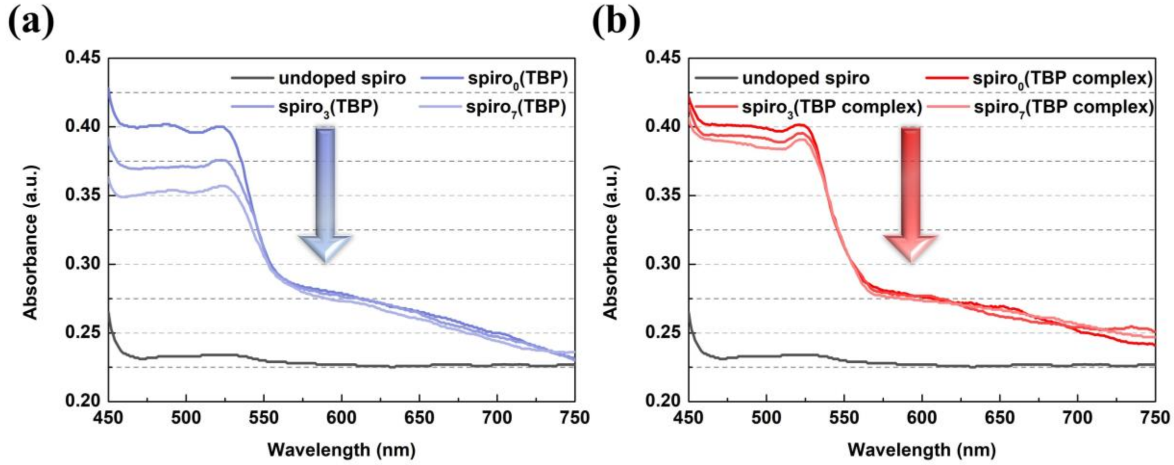

Fig. S18 Absorption spectra of the different spiro solutions with **a** TBP and **b** TBP complex

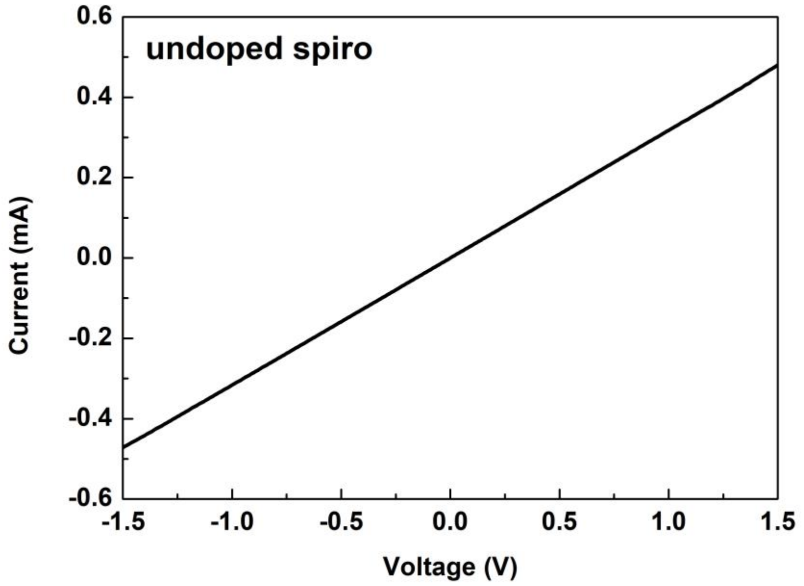

Fig. S19 *I-V* curves of the device with the structure of ITO/undoped spiro/Ag

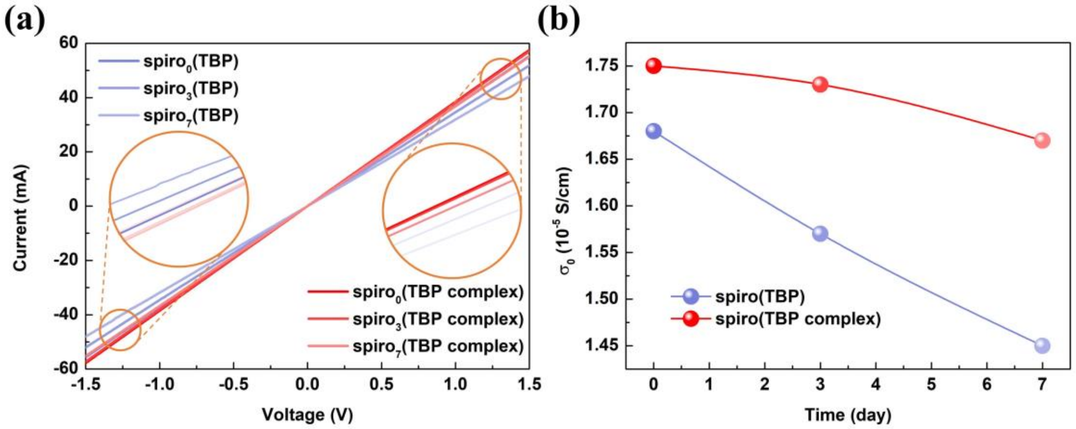

Fig. S20 a *I-V* curves of the devices with the structure of ITO/different spiro-based films/Ag. **b** Variation of the σ_0 of different spiro-based films with time

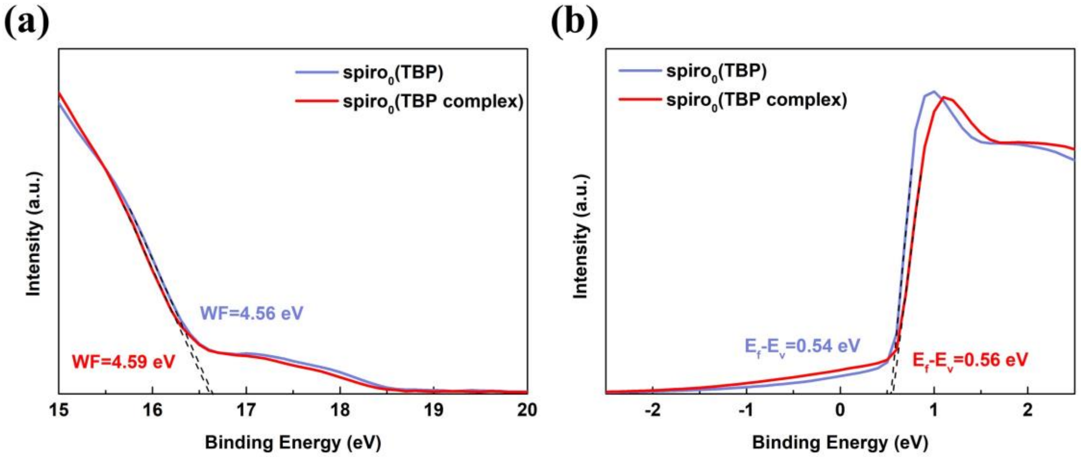

Fig. S21 UPS spectra exhibiting the **a** secondary-electron cut-off and **b** valence band (VB) region of the spiro$_0$(TBP) and spiro$_0$(TBP complex) films. The UPS measurement is commonly utilized to obtain the work function (WF) and the difference between the Fermi level (E_f) and the VB maximum for the film samples [S4]

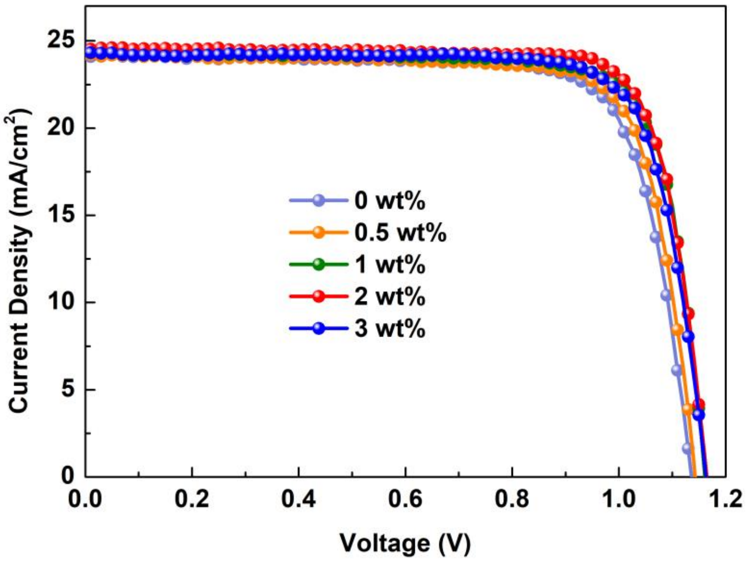

Fig. S22 *J-V* curves of the PSCs with different contents of 1,4-DITFB

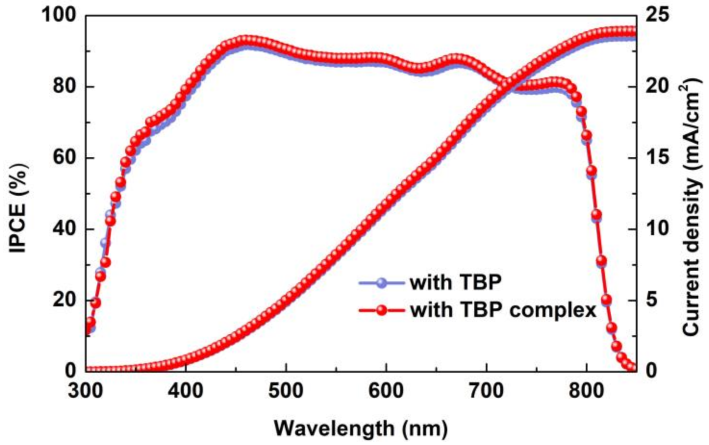

Fig. S23 IPCE spectra of the devices with TBP and TBP complex

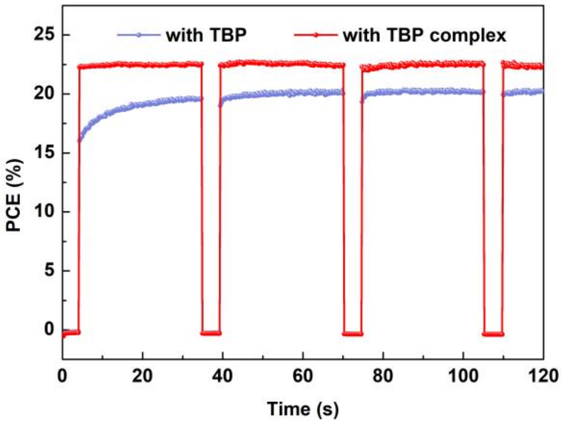

Fig. S24 PCE response tests of the devices with TBP and TBP complex

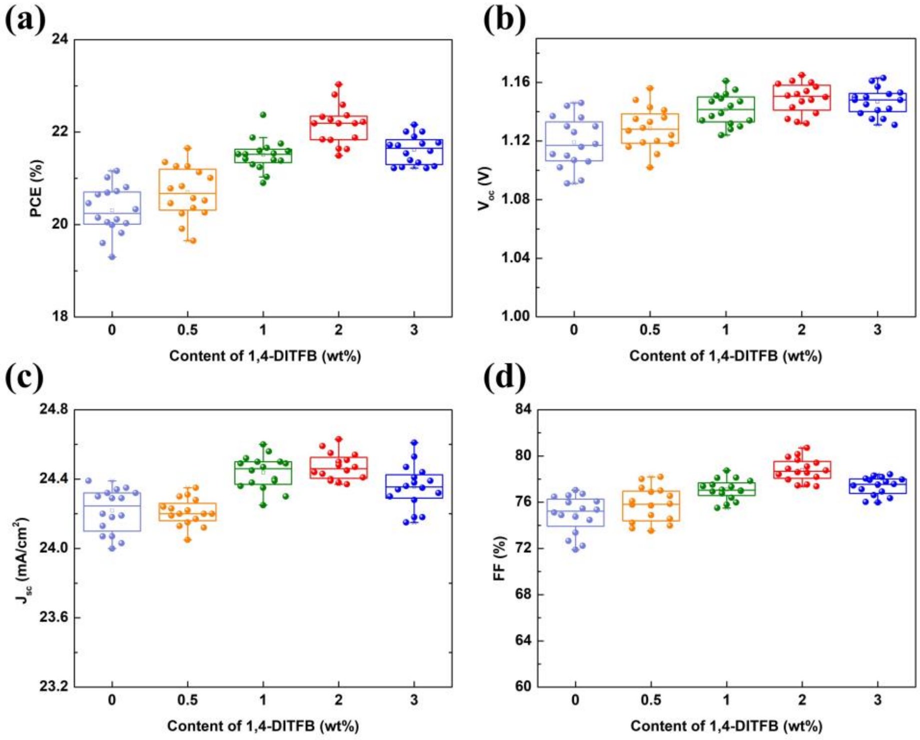

Fig. S25 Distribution of the **a** PCE, **b** V_{oc}, **c** J_{sc} and **d** FF of the devices with different content of 1,4-DITFB (16 individual cells are included)

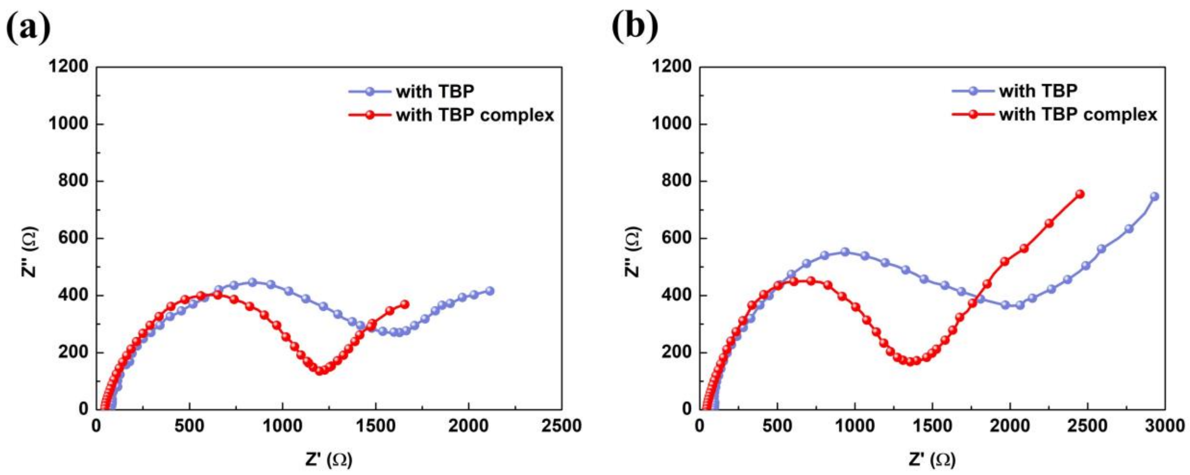

Fig. S26 Nyquist plots of the devices with TBP and TBP complex measured under **a** 0.6 V bias voltage and **b** 0.4 bias voltage

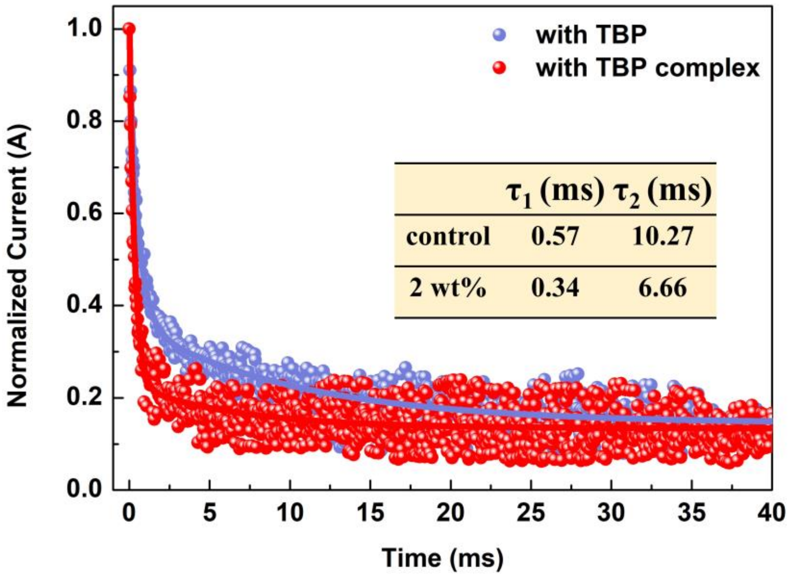

Fig. S27 TPC based on the devices with TBP and TBP complex

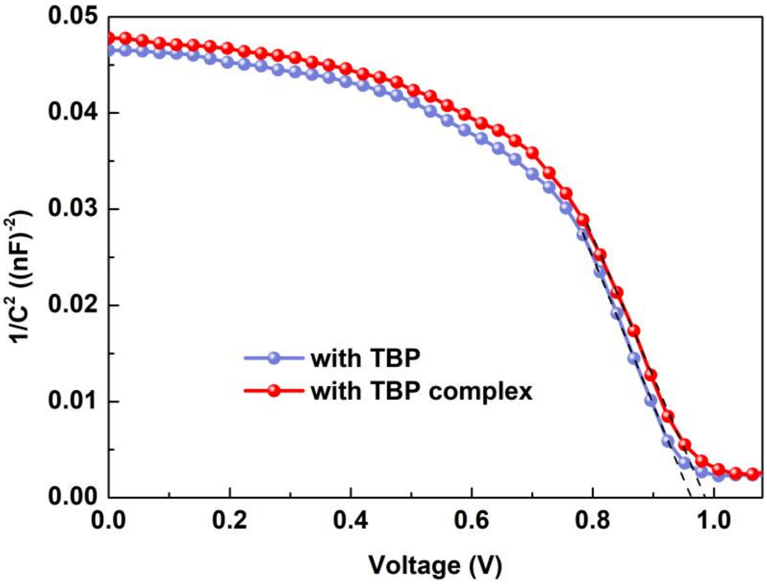

Fig. S28 *C-V* characteristic curves of the devices with TBP and TBP complex

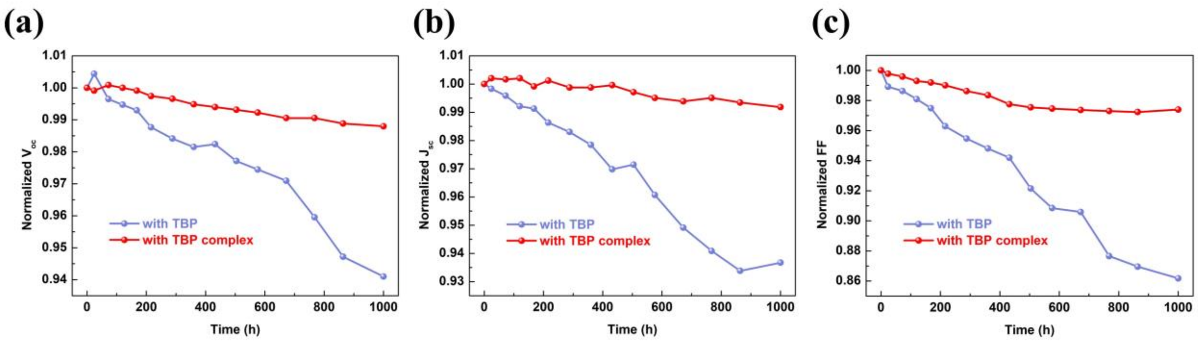

Fig. S29 a V_{oc}, **b** J_{sc} and **c** FF evolution of the devices with TBP and TBP complex without encapsulation in N_2 atmosphere at room temperature

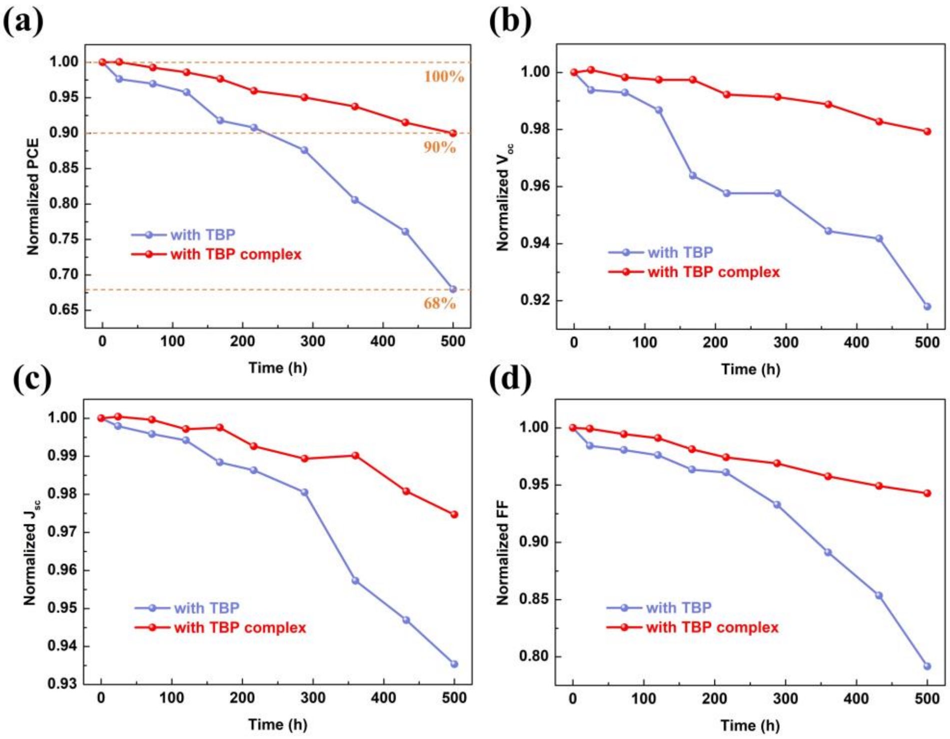

Fig. S30 a PCE, **b** V_{oc}, **c** J_{sc} and **d** FF evolution of the devices with TBP and TBP complex without encapsulation in 40 ± 5% RH at room temperature

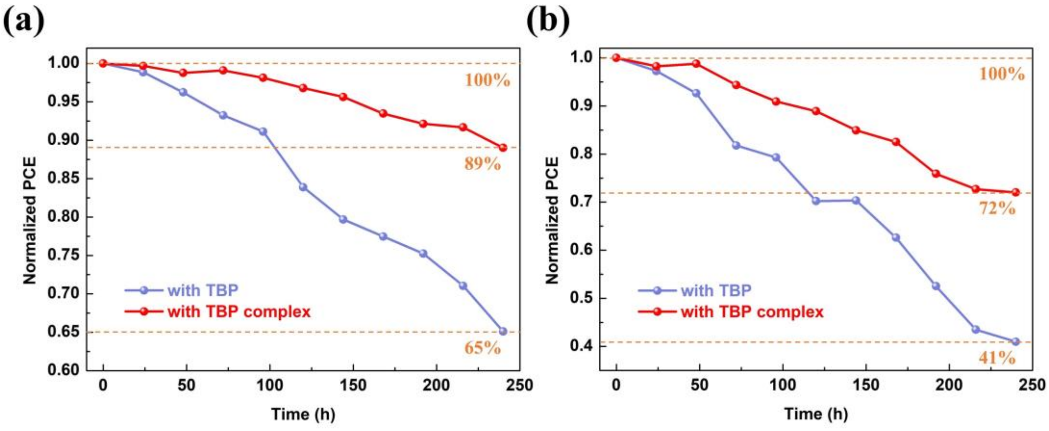

Fig. S31 PCE evolution of the devices with TBP and TBP complex without encapsulation in **a** 60 ± 5% RH and **b** about 85% RH at room temperature

In stability tests, the Ag electrode was replaced with the Au electrode to exclude the influence of I^- ions migration and their reaction with Ag [S5].

Table S1 The photovoltaic parameters of the PSCs with normal TBP, triple TBP and triple TBP complex measured under different scanning directions, including open-circuit voltage (V_{oc}), short-circuit current density (J_{sc}), fill factor (FF) and PCE

Device	scanning direction	V_{oc} (V)	J_{sc} (mA/cm^2)	FF (%)	PCE (%)
normal TBP	reverse	1.118	24.05	77.10	20.73
	forward	1.102	24.01	76.01	20.11
triple TBP	reverse	1.141	22.78	65.60	17.05
	forward	1.133	22.77	62.25	16.06
triple TBP complex	reverse	1.154	23.36	73.66	19.86
	forward	1.152	23.29	72.34	19.41

Table S2 The variation of the σ_0 of different spiro-based films with time

Time (day) / σ_0 (s/cm) / sample	0	3	7
spiro	1.68×10^{-5}	1.57×10^{-5}	1.45×10^{-5}
spiro(1,4-DITFB)	1.75×10^{-5}	1.73×10^{-5}	1.67×10^{-5}
undoped spiro	1.44×10^{-7}	1.44×10^{-7}	1.44×10^{-7}

Table S3 The champion photovoltaic parameters of the PSCs with different content of 1,4-DITFB

Content (wt%)	V_{oc} (V)	J_{sc} (mA cm^{-2})	FF (%)	PCE (%)
0	1.136	24.18	77.05	21.16
0.5	1.143	24.22	78.19	21.65
1	1.161	24.47	78.74	22.37
2	1.165	24.50	80.70	23.03
3	1.163	24.38	78.17	22.16

Table S4 The photovoltaic parameters of the champion PSCs with TBP and TBP complex

Device	scanning direction	V_{oc} (V)	J_{sc} (mA cm^{-2})	FF (%)	PCE (%)	HI (%)	J_{sc} from IPCE
with TBP	reverse	1.136	24.18	77.05	21.16	4.58	23.56
	forward	1.116	24.15	74.92	20.19		
with TBP complex	reverse	1.165	24.50	80.70	23.03	1.09	23.90
	forward	1.162	24.50	80.03	22.78		

Supplementary References

[S1]T. Matsui, T. Yamamoto, T. Nishihara, R. Morisawa, T. Yokoyama et al., Compositional engineering for thermally stable, highly efficient perovskite solar cells exceeding 20% power conversion efficiency with 85 °C/85% 1000 h stability. Adv. Mater. **31**(10), 1806823 (2019). https://doi.org/10.1002/adma.201806823

[S2]S.Y. Lee, B.H. Boo, Density functional theory study of vibrational spectra of fluorene. J. Phys. Chem. **100**(21), 8782-8785 (1996). https://doi.org/10.1021/jp960020g

[S3]S. Yurdakul, M. Bahat, Fourier transform infrared and Raman spectroscopic studies on 4-tert.-butylpyridine and its metal(II) tetracyanonickelate complexes. J. Mol. Struct. **412**(1),

97-102 (1997). https://doi.org/10.1016/S0022-2860(96)09392-1

[S4] X. Liu, Z. Liu, B. Sun, X. Tan, H. Ye et al., 17.46% efficient and highly stable carbon-based planar perovskite solar cells employing Ni-doped rutile TiO_2 as electron transport layer. Nano Energy **50**, 201-211 (2018). https://doi.org/10.1016/j.nanoen.2018.05.031

[S5] Y. Kato, L.K. Ono, M.V. Lee, S. Wang, S.R. Raga et al., Silver iodide formation in methyl ammonium lead iodide perovskite solar cells with silver top electrodes. Adv. Mater. Interfaces **2**(13), 1500195 (2015). https://doi.org/10.1002/admi.201500195
